# Decoding Decisions: Personality-Interest Motivational Sequences as Predictors of Career Paths

**DOI:** 10.12688/f1000research.176767.1

**Published:** 2026-02-10

**Authors:** Gary Clifford Townsend, Portia Webb

**Affiliations:** 1 Faculty of Humanities, Independent Institute of Education, Emeris, Port Elizabeth, Eastern Cape, 6001, South Africa; 2 Faculty of Commerce, Independent Institute of Education, Emeris, Port Elizabeth, Eastern Cape, 6001, South Africa

**Keywords:** PIMS, motivational sequences, personality traits, vocational interests, career development, RIASEC, TPQ

## Abstract

**Background:**

Research on personality and vocational interests has reached a plateau, with correlations stabilising around *r* ≈ .48. This plateau occurs because dominant models, such as Holland’s RIASEC (Realistic, Investigative, Artistic, Social, Enterprising, Conventional) theory, treat interests as fixed matches to environments, rather than as dynamic outcomes of an individual’s psychology. This study introduces the Personality-Interest Motivational Sequences (PIMS) framework, which reinterprets vocational interests as emergent properties of facet-level personality trait configurations.

**Methods:**

We analysed data from 504 final-year South African university students assessed with the Townsend Personality Questionnaire (TPQ) and the O*NET Interest Profiler. A multinomial logistic regression model, trained on a subset of 404 participants and tested on a holdout set of 100, was used to predict primary RIASEC categories from 30 TPQ facet-level traits.

**Results:**

The PIMS model predicted primary RIASEC categories with 60.0% accuracy, substantially exceeding the chance level of 16.7%. Investigative interests were predicted most effectively, with distinct facet-level sequences identified for this and other types. For example, an Investigative profile was characterised by low Resilience and Sociability facets coupled with high Thrill-seeking and Competence. Notably, the model found no single, consistent personality profile for Social interests, which reveals potential heterogeneity within this RIASEC category.

**Conclusions:**

The findings support the PIMS framework, demonstrating that vocational interests are systematic outcomes of facet-level personality architecture. This moves the field from describing static correlations to modelling generative psychological mechanisms. The framework provides a foundation for moving beyond typological matching towards dynamic, personalised career pathwaying.

## 1. Introduction

The relationship between personality and vocational interests represents a key area of research within vocational psychology, yet one characterised by a notable lack of theoretical progress. This theoretical deadlock is particularly interesting given the strong influence of John L. Holland’s (1958) theory of vocational personalities, which, for over half a century, has provided the dominant model for conceptualising this relationship ( Nauta, 2010). Studies consistently show that this stagnation manifests as a persistent ceiling effect, with meta-analyses having reported correlations between personality and vocational interests stabilising around *r* ≈ .48 ( Larson et al., 2002; Staggs et al., 2007). This limitation shows that, while a reliable association exists, the fundamental understanding of why this is the case still remains somewhat incomplete. This is noteworthy because vocational interests present as persistent trait-like dispositions that are shown to correlate with life outcomes, including career choice, educational performance, and long-term career success ( Nye, et al., 2017; Rounds & Su, 2014; Low, et al., 2005; Strong, 1943).

In light of these relationships, the inability of the field to move beyond broad domain-level correlations–to explain the very source of these interests–remains a significant theoretical obstacle. This limitation, we argue, originates from a failure to disaggregate personality traits from environmental categories within traditional typological frameworks. As a result of this, the field is left with descriptive models that can identify what interests align with which personalities, but not how or why these interests surface from an individual’s psychological architecture.

### 1.1 The personality-interest conflation problem

The ongoing absence of theoretical progress in linking personality and vocational interests stems from a basic conceptual conflation within the dominant typological model. While Holland’s (1997) proposition–that vocational interests are an expression of personality–successfully organised the field, it also had the unintended consequence of theoretically blurring the two constructs. This conflation directly influenced both how researchers conceptualised these constructs in practice and the way they measured them. Understandably, most of this practice occurred within a historical context that lacked robust, separate models for either construct ( Larson, et al., 2002), which allowed typologies based on strained ideas like ‘behavioural styles’ ( Marston, 1928) or ‘psychological types’ ( Jung, 1971) to persist as the nexus between personality and interests as though they were isomorphic. Despite this, Holland’s RIASEC model emerged as a pragmatic and accessible solution ( Costa et al., 1984; Nauta, 2010), ultimately cementing a framework where environmental categories (e.g., ‘Social’ work settings) are defined using personality descriptors (e.g., ‘helpful, cooperative’) and vice-versa.

However, over time the persistent moderate relationships ( *r* ≈ .48)–confirmed by meta-analyses–eventually persuaded the field to accept that while related, personality traits and vocational interests are more than likely distinct constructs ( Larson, et al., 2002; Tokar et al., 1998, Wille, et al., 2025). Notwithstanding the theoretical acceptance of this distinction, Holland’s dominant theoretical framework–that depends on the congruence (strong fit) between personality and work environment–persists to date ( Etzel, et al., 2025, Wille, et al., 2014).

More importantly for this research, the conflation has tangible practical costs, as it obscures the unique underlying predictive relationships of these unique constructs ( Wei, 2024). The core issue here was highlighted in an earlier finding by De Fruyt and Mervielde (1999), where they demonstrated that–as opposed to RIASEC types–Big Five traits predict employment status much better while these types, in turn, predict the nature of employment better than traits. This clear separation suggests that the typological model’s merging of environmental categories with personality structure most likely constrains the explanatory and predictive power of the respective independent constructs ( Wei, 2024).

### 1.2 Breaking the correlation plateau

Attempts to progress beyond the correlation plateau have consistently pointed to the need for a more systemic, mechanistic framework Etzel, et al. (2025). Researchers like Costa, McCrae, and Holland (1984) observed that even their own methodologically sophisticated attempt to avoid item-content overlap produced correlations with obvious theoretical boundaries. It was clear from their observations that key RIASEC types implied there were personality dimensions, which their contemporary model could not capture ( Costa & McCrae, 1995). More recently, Veres and Póka (2024) were confronted with a similar frustration when correlating HEXACO personality domains with RIASEC vocational interests ( *r* ≈ .45). Even their attempts at a predictive hierarchical regression exercise resulted in rather tenuous RIASEC *R ^2^* values of .14, .04, .24, .26, .17, and .13 respectively ( Veres & Póka, 2024). While explanatory, these approaches hit statistical ceilings because they are attempting to quantify systems that are basically heterogenous. These insights point to the need for a more granular, facet-level, personality approach to explain interest aetiology–as opposed to approaches that simply catalogue motivational themes–without explaining their origins from an individual’s psychological architecture. To date, this body of work leaves a critical explanatory gap between broad personality domains and expressed vocational interests ( Nauta, 2010).

The PIMS framework fills this gap by modelling generative psychological mechanisms. This approach proposes that vocational interests are dynamic outcomes of specific, facet-level personality trait configurations, thereby moving from modelling correlation (cf. Etzel, et al., 2025) to proposing a causative framework. It directly answers the historical need, presaged by Costa et al. (1984), for a model capturing the nuanced personality facets underlying interests. Operationalised with a detailed facet-level measure–the Townsend Personality Questionnaire (TPQ; Townsend, 2017)–the PIMS framework tests the proposition that interests are manifestations of multi-faceted motivational sequences, and methodologically disentangles personality causes from environmental interest outcomes. This provides a mechanistic model of interest origin, which addresses the longstanding call to move beyond typological limitations ( Larson et al., 2002; McCrae et al., 2000; Nauta, 2010).

### 1.3 The PIMS framework: A methodological resolution

**Figure 1.**
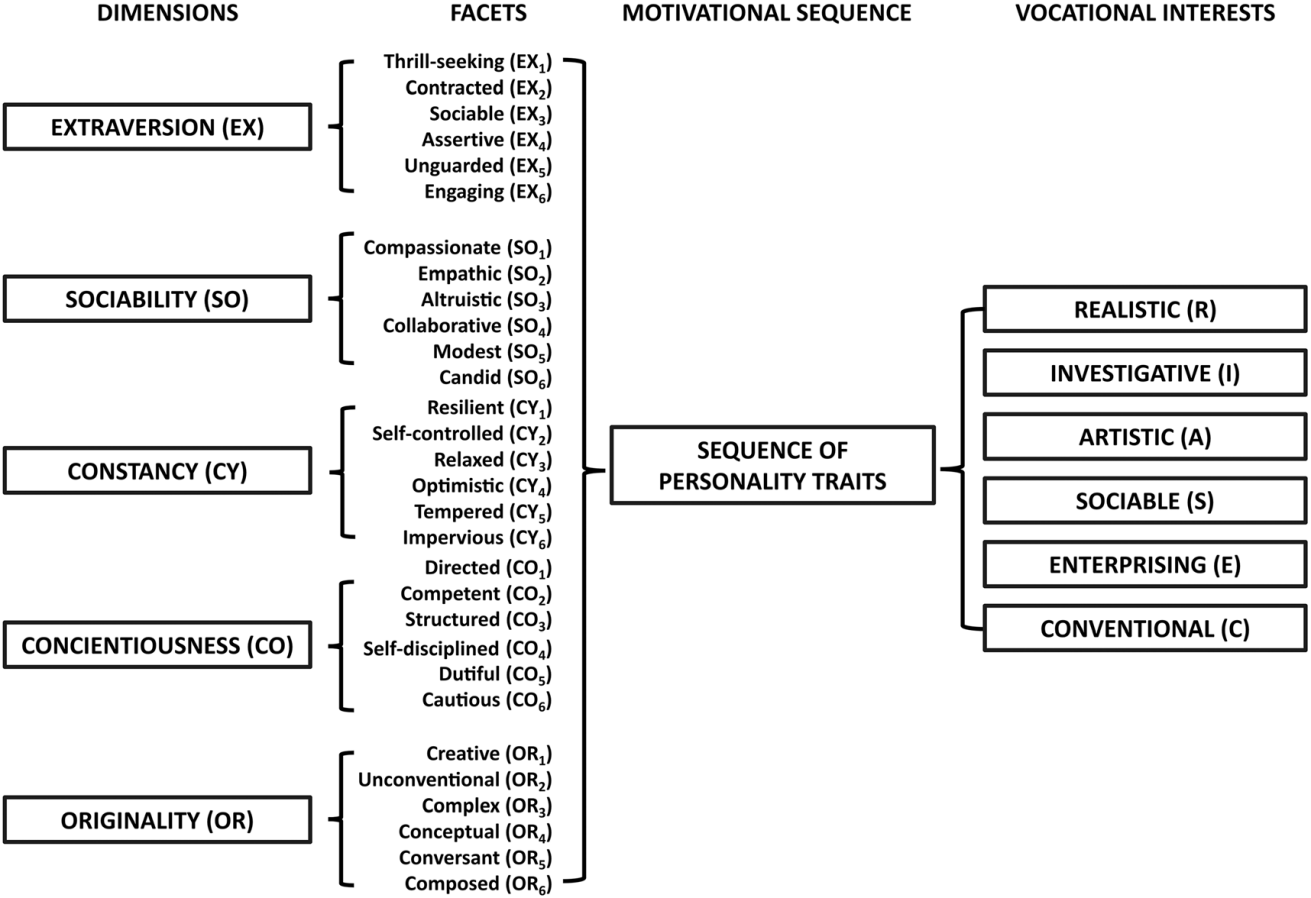
The Personality-Interest Motivational Sequences (PIMS) model. **Note:** This conceptual model outlines the proposed dynamic process of vocational interest formation. It shows how facet-level personality traits combine into motivational sequences, which in turn predispose individuals towards specific vocational interest categories (e.g., RIASEC types). The framework represents a paradigm shift from static typological matching to a dynamic model of personality-driven pathwaying.

Having diagnosed the persistent explanatory plateau and its roots in the personality-interest conceptual conflation, the necessary next step is to propose a framework that methodologically disentangles these constructs. To address this problematic conflation, the PIMS model explicitly suggests that vocational interests are not static environmental alignments but, are dynamic outcomes of an individual’s personality architecture. This new conceptual framework integrates three core elements: empirically measured personality traits, the facet-level motivational sequences they form, and the subsequent alignment of these sequences with specific vocational interests (See Figure 1).

The PIMS framework illustrates how dynamic interactions between facet-level personality traits form motivational sequences that predispose individuals toward specific vocational interest environments, moving beyond static RIASEC or other typologies. This research proposes that we look at interests not as environmental mirrors but as emerging properties of personality–a shift from static taxonomies to dynamic, individualised pathways. This new approach is designed to resolve a fundamental methodological circularity inherent in the typological approach. It does away with the need to arbitrarily assign personality descriptors to RIASEC environmental preferences retroactively.

The inherent variability of the RIASEC structure highlights this issue, which even Holland and Gottfredson (1992) described as a ‘misshapen polygon’, a concession supported by empirical evidence showing the data often better fit a circumplex rather than a perfect hexagon ( Armstrong, et al., 2003; Nauta, 2010). Nevertheless, researchers often persist in forcing this irregular model into an inflexible circumplex, a pursuit that prioritises geometric elegance over psychological accuracy (cf. Etzel, et al., 2025). In direct contrast, our framework treats Holland’s categories strictly as broad vocational environments, not as personality equivalents. By methodologically decoupling these environmental categories from personality constructs, the PIMS model opens new avenues for multidimensional career guidance strategies that go beyond static typologies and–for the future exploration of interest taxonomies–beyond RIASEC’s environmental categories. In summary, this methodological decoupling is the essential first step that allows for the investigation of interests as effects of personality, rather than their conceptual redefinition.

### 1.4 Proposing a new, explanatory framework

The PIMS model serves a different, more fundamental purpose than established motivational inventories such as the Motivational Appraisal of Personal Potential (MAPP), Schein’s (1990) Career Anchors, or Super’s (1970) Work Values Inventory. While these inventories, among others, are valuable for classifying the manifestations of motivation–such as the need for autonomy or the desire to serve–they are largely descriptive. They identify motivators without explaining their psychological origins. Alternatively, the PIMS model addresses this explanatory gap by proposing a generative mechanism in which facet-level personality traits interact in dynamic sequences to produce these broader motivational themes. For example, ‘Persistence’ ( Rounds & Su, 2014) is no longer merely a primary cause but an outcome: a narrative constructed from an underlying profile of higher Constancy (Resilience [CY _2_] and Tempered [CY _6_] facets) and higher Conscientiousness (Self-disciplined [CO _1_], Dutiful [CO _2_], and Directed [CO _6_] facets). In this way, PIMS provides a personality-based theory of the engine, rather than merely cataloguing the vehicles it powers.

To translate this theoretical engine into a testable model, a specific methodological shift was required, building on the decoupling described in section 1.3. This entailed prioritising the separation of personality traits and environmental interests as constructs to be able to answer the central question: if interests are ‘traitlike preferences’ ( Rounds & Su, 2014, p. 1), why are models like RIASEC unable to reliably connect interests and personality in a clear, consistent way? We assert that the core problem comes not just from confusing personality traits with personality types but the unwitting practice of treating self-reported interests as input components to what motivates (e.g., direction, vigour, persistence, etc.) goal-oriented behaviours and subsequent goal-attainment ( Rounds & Su, 2014; Low et al. 2005).

By disentangling these elements–measuring personality traits independently, without linking them to environmental interests–we avoid forcing interests into neat hexagonal categories. This makes it possible to observe how the nuances of someone’s personality actually steer them toward specific career directions, as opposed to just assigning people to interest categories based on a particular activity or object of interest.

**Figure 2.**
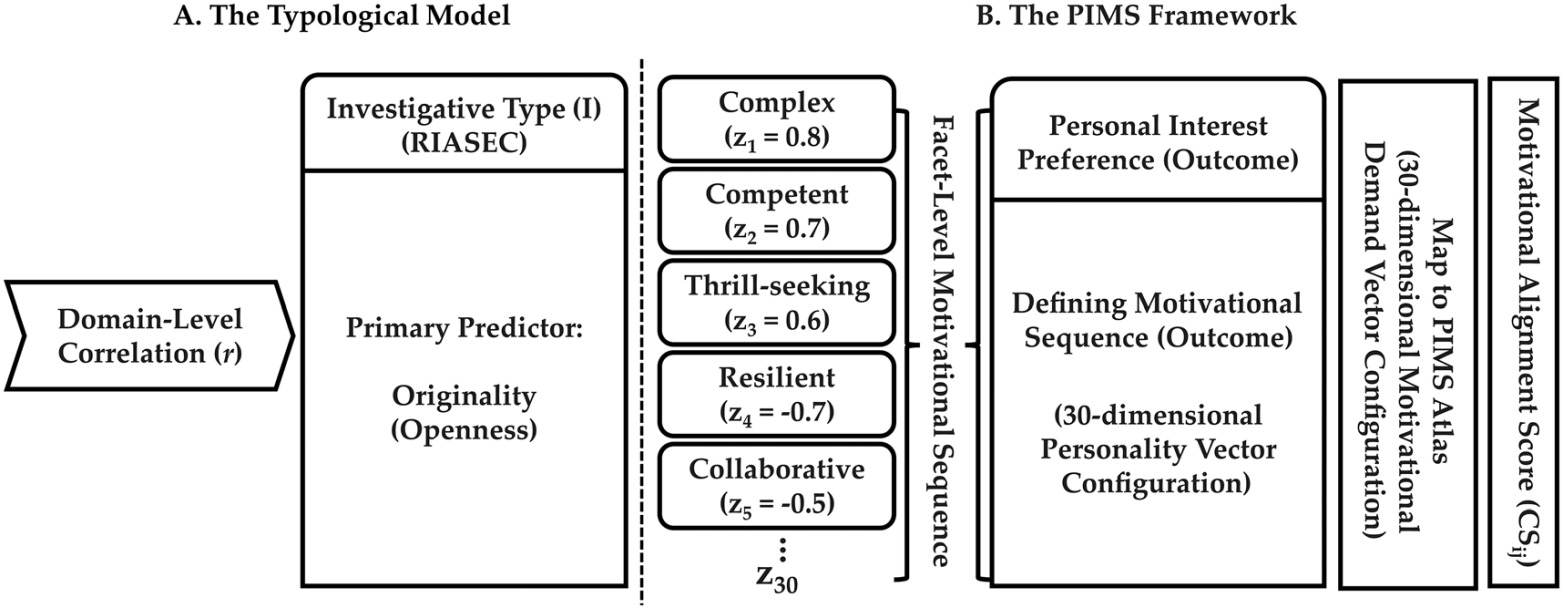
Model comparison of the typological and PIMS frameworks. **Note:** This figure illustrates the paradigm shift from the Typological approach (A) to the Personality-Interest Motivational Sequences (PIMS) framework (B). The traditional model treats interests as static categories aligned with broad personality domains (Extraversion; Sociability [Agreeableness]; Constancy [inverse of Neuroticism]; Conscientiousness; Originality [Openness]). The PIMS model reconceptualises them as dynamic outcomes of specific, facet-level trait interactions, using the Investigative type as a specific exemplar of this generative mechanism. In practice the individual’s standardised scores across the 30 TPQ facets form a 30-dimensional personality vector configuration. This personal configuration is algorithmically compared against the 30-dimensional motivational demand vector configuration for each occupation within the PIMS Atlas, yielding a congruence coefficient via cosine similarity.

The PIMS framework suggests that vocational interests are not simple reflections of major personality domains, nor do they just align with fixed environmental categories. Rather, interests develop from something more complex and dynamic: the way unique personality facets combine. Also, these facets do not function as isolated scores; instead, they form motivational sequences–patterns of relative strengths and weaknesses–that guide individuals toward particular sets of interests. For example, an Investigative interest is not merely predicted by higher Openness (TPQ; Originality), but by a precise sequence involving lower Resilience, lower Sociability facets, and higher Thrill-seeking. By focusing on these granular, empirically derived sequences, the PIMS model provides a generative mechanism for interest formation, thereby directly addressing the conflation and heterogeneity ( Etzel, et al., 2025) that have limited earlier typological approaches (See Figure 2 for a conceptual comparison).

To operationalise this framework, this study used the Townsend Personality Questionnaire (TPQ; Townsend, 2017) for its ability to assesses 30 facet-level traits across the Big Five dimensions. The researchers applied a multinomial regression model to identify which of the 30 traits actually predict preferences for each RIASEC environment. They did not rely on assumptions about which links mattered but allowed the data to guide the process. That is the foundation of the PIMS model. Its core proposition is that career guidance emerges not from isolated traits, but from their dynamic configuration and interaction. In this framework, personality facets do not simply follow career decisions, they actively influence them. This is the focus of the research at the core of the PIMS model: specific patterns of traits come together to form broader motivational drives that result in significant outcomes ( Veres & Póka, 2024).

Consequently, to empirically test this proposition, this study was designed to answer the following research questions:

• Which facet-level personality traits form predictive personality-interest motivational sequences (PIMS) for each of Holland’s RIASEC vocational-interest environments?
• To what extent does the PIMS model, using facet-level traits, improve the prediction of career interest preferences over domain-level trait approaches and the RIASEC typology?
• How does the PIMS framework explain heterogeneity within broad RIASEC types?

## 2. Methods

This study employed a quantitative, cross-sectional design to empirically test the proposition that vocational interests are emergent properties of underlying personality trait configurations. The primary aim was to investigate whether facet-level personality traits could predict primary vocational interest categories, thereby operationalising the proposed Personality-Interest Motivational Sequences (PIMS) framework.

### 2.1 Participants

In this study 504 final-year university students were randomly recruited from campuses across South Africa. This population was specifically targeted because their approaching career transition made them ideal for testing a model designed to predict career paths. The sample itself was diverse. It comprised 37.7% male, 61.5% female, and 0.8% non-binary participants. The majority were aged between 21 and 25 (70.9%), with 23.1% in the 14-20 range, 4.8% between 26 and 30, and only 1.2% older than that. Regarding education, most had completed high school (66.8%), 14% had attended college or technical college, 18.4% already held university degrees, and a small fraction (0.8%) possessed postgraduate qualifications. Among all participants, 30.6% identified as Black, 17.8% as Coloured, 9.7% as Indian, and 41.9% as White. Their academic majors ranged from Business Management (10.3%), Accounting (9.7%), Psychology (16.8%), Programming (17.2%), Criminology (5.7%), Education (10.1%), along with several other fields.

### 2.2 Measures

2.2.1 Personality measure

The Townsend Personality Questionnaire (TPQ; Townsend, 2017) is a 120-item assessment based on the Five-Factor Model and measures five core personality dimensions namely, Extraversion, Sociability (Agreeableness), Constancy (Inverse of Neuroticism), Conscientiousness, and Originality (Openness). While the TPQ uses its own nomenclature for dimensions and facets such as ‘Sociability’ for Agreeableness, ‘Constancy’ for Neuroticism, and ‘Originality’ for Openness its model is by design aligned with the established Five-Factor Model. This ensures the measure captures the consensual broad domains of personality, while its specific facet labels are designed to reflect motivational, non-stigmatising language suited to applied contexts ( Townsend & Web, 2026). Each dimension has six underlying facets, giving a detailed 30-facet profile. To reduce response bias, 48% of the items are reverse-scored, and all items are presented in a randomised order. In practice, raw scores are standardised into Sten scores (Standard Ten; mean = 5.5, SD = 2) for interpretation.

2.2.2 Vocational interest measure

Consistent with the study’s theoretical decoupling of personality from environmental typology, vocational interests were measured using the O*NET Interest Profiler ( Lewis & Rivkin, 1999; Rounds et al., 1999a), which aligns with Holland’s (1997) RIASEC typology. This instrument measures the six RIASEC categories–Realistic, Investigative, Artistic, Social, Enterprising, Conventional–defining them by their characteristic activities, competencies, and environmental rewards rather than by personality descriptors. It uses a mixed-item presentation format, where questions are randomised rather than grouped by RIASEC domain, to mitigate response biases such as acquiescence and to enhance ecological validity by encouraging respondents to evaluate each item independently.

### 2.3 Psychometric properties

2.3.1 O*NET interest profiler

The O*NET Interest Profiler has solid internal consistency, with Cronbach’s α ranges from.81 for the short form ( Rounds et al., 1999b) to.93–.96 for individual RIASEC scales ( Nauta, 2010). In this study, the instrument demonstrated strong internal consistency (α = .75) as well. Its development and validation support its application in career decision-making situations, such as assisting university students in selecting their majors ( Nauta, 2010). This makes it suitable for our research.

2.3.2 Townsend Personality Questionnaire (TPQ)

Assessments across multinational samples ( *N* = 1,050) using the Townsend Personality Questionnaire (TPQ) have shown robust internal consistency (α = .70–.87). A parallel form’s reliability test against Goldberg’s IPIP Five-Factor Marker scale ( Goldberg, 1992, 1999; Goldberg et al., 2006) produced solid correlations ( *r* = .72–.84), and a subsequent test-retest reliability exercise over four weeks showed good stability ( *r* = .70–.87). Furthermore, Rasch analysis (WINSTEPS 3.92.1) confirmed strong measurement properties. Item INFIT/OUTFIT mean squares fell within the optimal range of 0.6–1.4 for Likert scales, confirming essential unidimensionality. The 4-point rating scale functioned effectively. Thresholds progressed sequentially, and the average measures were aligned as anticipated. Item separation reliability was high (G ranged from 6.78 to 8.06, with reliability at.98 across all dimensions). Person separation reliability, while marginally lower, was still within an acceptable range (G = 1.37–2.39; Reliability = .65–.85). This confirms the instrument can differentiate between respondents with different levels of the trait ( Townsend, 2017; Townsend & Nazare, 2023). Given its Rasch validation, the TPQ’s measurement properties are sample-independent, which supports its robustness as a measure of Five-Factor Model traits in this context.

### 2.4 Procedure

The Townsend Personality Questionnaire ( Townsend, 2017) and the O*NET Interest Profiler ( Lewis & Rivkin, 1999; Rounds et al., 1999a) were hosted on a secure server. Each participant was sent an invitation to participate via email which included the access link to the online questionnaires. The online system limited respondents to one response per question but allowed them to adjust their selections until final submission. This study was approved by the IIE Ethics Committee, Independent Institute of Education, Emeris (R.1602223 [REC]). Written consent for the primary questionnaires was obtained; participants received an email with the link to the questionnaires outlining the study’s purpose, anonymity, and voluntary nature, with survey submission constituting consent. Conversely, explicit consent for the optional longitudinal component was obtained via a dedicated opt-in field where participants voluntarily provided their email addresses for future contact. JASP (JASP Team, 2024) and R ( R Core Team, 2025) were used for statistical analysis and visualisation.

### 2.5 Analysis

The analytical approach was constructed to directly test the core propositions of the PIMS framework. We used a multinomial logistic regression model to predict each participant’s dominant RIASEC vocational interest category based on their thirty facet-level scores from the TPQ. To ensure we could generalise our findings as well as avoid an overfitting model, we divided the total data set into a training set of 404 participants for the model development and kept aside a test set of 100 randomly selected participants that we used exclusively for the final evaluation ( James et al., 2021). This holdout validation strategy provides a rigorous, unbiased estimate of the model’s real-world predictive performance. Given the exploratory nature of this research, and the large number of predictors we were using (thirty facets in total), we decided to use a significance level of α = .10 to avoid potential Type II errors. This is a recognised strategy in similar research contexts ( Gelman & Carlin, 2014). So, considering this is a hypothesis-generating research exercise, to ensure that the facet-level sequences we identified were both statistically indicative and conceptually meaningful we broadened our final selection of the predictors to include a combination of their effect size (standardised beta coefficient, *β*), their theoretical plausibility within the Five-Factor Model, as well as their contribution to the model’s overall predictive accuracy ( Amrhein et al., 2019; Wasserstein & Lazar, 2016).

## 3. Results

The analysis sought to answer the primary research questions by first establishing the overall predictive power of the facet-level approach and then uncovering the specific personality-interest motivational sequences (PIMS) underlying vocational preferences. The findings show compelling empirical support for the PIMS framework–supporting the idea that vocational interests are systematic outcomes of an individual’s facet-level personality architecture. The results are presented in three key parts: the model’s overall accuracy, its differential performance across interest types, and the identification of the core motivational sequences themselves.

### 3.1 Overall model predictive accuracy

The multinomial regression model built for predicting the primary RIASEC categories from 30 TPQ facets, showed significant predictive effectiveness. The model was applied to a held-out test set ( *n* = 100), achieving an overall accuracy of 60.0% (i.e. it accurately predicted the vocational interests of 60 out of 100 previously unobserved individuals) which substantially exceeded the 16.7% chance-level expected for a six-category outcome. This outcome provides preliminary but compelling evidence that systematic relationships exist between specific personality facet configurations and broad vocational interest patterns, which supports the central hypothesis of the PIMS framework.

### 3.2 Differential performance across interest types

The model’s performance, however, was not uniform across all RIASEC types, as detailed in Table 1. Investigative (I) interests emerged as the paradigm case for the PIMS model. The model was accurate for this type (precision of 76.5%) and identified almost all true positives (recall of 86.7%). In addition, substantial meaningful results were produced when testing the Artistic (A) and Conventional (C) interests, with Area Under the Curve (AUC) values above 0.76. Here, however, the model’s precision was slightly less effective in distinguishing Social (S; AUC = 0.767) and Enterprising (E; AUC = 0.694) interests, suggesting that the higher-order categories may contain more different underlying motivational sequences ( Nye et al., 2017). For Realistic (R) interests the model showed a very strong AUC (0.985), but this result is a statistical outlier due to the extremely low base rate of this interest category in our university sample ( *n* = 9; 1.8%), which precludes stable estimation and reliable interpretation ( Cohen, 1992). This finding itself is conceptually informative, suggesting the classic ‘Realistic’ designation may be poorly represented in a modern academic population, a point we revisit in the Discussion.

**Table 1.** Model performance metrics by RIASEC category (Test set, *n* = 100).

RIASEC category	Precision	Recall (Sensitivity)	F1 score	AUC
Investigative (I)	0.765	0.867	0.813	0.837
Conventional (C)	0.706	0.667	0.686	0.803
Artistic (A)	0.632	0.706	0.667	0.769
Social (S)	0.545	0.545	0.545	0.767
Enterprising (E)	0.455	0.385	0.417	0.694
Realistic (R)	0.333	0.500	0.400	0.985
Average/Total	0.594	0.600	0.595	0.809

**Note.** Performance metrics evaluated on the held-out test set (n = 100). Precision: proportion of correctly identified positive predictions. Recall (Sensitivity): proportion of actual positives correctly identified. F1 Score: harmonic mean of Precision and Recall (range 0–1, higher is better). AUC: Area Under the Receiver Operating Characteristic Curve, measuring overall discriminative ability (range 0.5–1, where 1 denotes perfect classification).

Furthermore, a closer look at the confusion matrix revealed theoretically coherent patterns of misclassification. For instance, the model most often confused Enterprising and Social types. This made sense considering their adjacent positions on Holland’s hexagon and that they both focus on people-oriented areas ( Holland, 1997; Armstrong, et al., 2008). It is likely that the model was simply reflecting the fuzzy boundaries between these constructs within the RIASEC model rather than a failure of the predictive model, reinforcing the need for a more refined, personality-driven approach.

### 3.3 Personality-Interest Motivational Sequences (PIMS)

**Figure 3.**
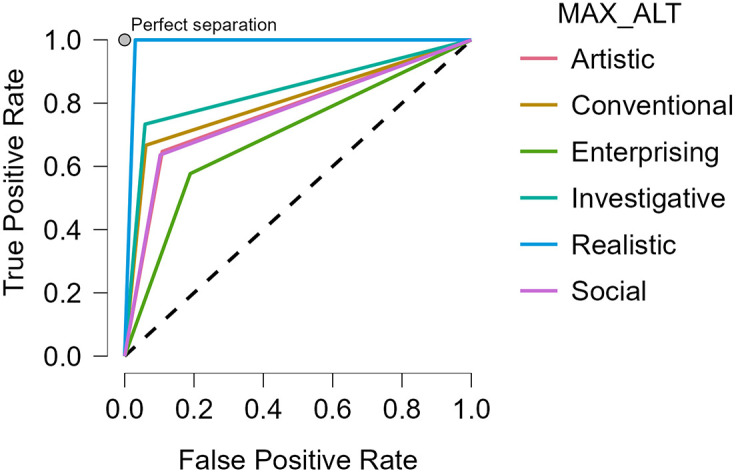
Receiver Operating Characteristic (ROC) curves for the multinomial regression model predicting RIASEC types on the held-out test set ( *n* = 100). **Note:** This figure presents the one-vs-rest ROC curves for each RIASEC vocational interest category, illustrating the diagnostic ability of the Personality-Interest Motivational Sequences (PIMS) model across all classification thresholds. The diagonal dashed line represents chance-level performance (Area Under the Curve [AUC] = 0.50), where the model offers no discriminatory power. Interpretation: Each curve plots the true positive rate (sensitivity) against the false positive rate (1–specificity). A curve that arches sharply towards the top-left corner indicates superior predictive performance for that specific interest type. The corresponding AUC value (see Table 1) quantifies this performance, where an AUC of 1.0 denotes perfect prediction and 0.5 denotes chance.

The heart of PIMS really comes to light in the regression coefficients, which break down exactly which personality traits are connected to getting people interested in each interest type. The analysis turned up a set of unique predictors for each RIASEC category, and this was not simply about a list of the things people are interested in, but about how those things actually motivate them, as laid out in Table 2. This particular set of personality–interest motivational sequences (PIMS) are seen in a whole new light when you look at Figure 3, which provides a clear picture of the main findings.

**Table 2.** Significant personality facet predictors of RIASEC interests.

RIASEC type	Predictor facet	*β*	*p*	Domain representation
Artistic (A-CIP)	Lower Altruistic	-0.478	<.05	Sociability (-)
	Lower Dutiful	-0.469	<.05	Conscientiousness (-)
	Higher Complex	0.317	<.10	Originality (+)
Conventional (C-CIP)	Lower Cautious	-0.747	<.05	Conscientiousness (-)
	Higher Tempered	0.707	<.05	Constancy (+++)
	Higher Composed	0.615	<.05	Constancy/Originality (+)
Investigative (I-CIP)	Lower Resilient	-1.073	<.001	Constancy (---)
	Lower Unguarded	-0.580	<.05	Sociability (--)
	Lower Collaborative	-0.486	<.05	Sociability (--)
	Higher Thrill-seeking	0.405	<.10	Extraversion (+)
	Higher Competent	0.439	<.10	Conscientiousness (+)
Realistic (R-CIP)	Higher Assertive	0.815	<.05	Extraversion (+++)
Enterprising (E-CIP)	Lower Contracted	-0.315	<.10	Extraversion (-)
	Higher Composed	0.385	<.10	Constancy/Originality (+)

**Note.** *β* = Standardised regression coefficient. Only facets significant at *p* < .10 are shown. The Domain Representation provides a qualitative interpretation of the facet’s effect size and direction based on the magnitude of *β*: (+) or (-) indicates a smaller-moderate effect; (++) or (--) indicates a moderate-strong effect; (+++) or (---) indicates the strongest effects observed. Some facets, such as ‘Composed,’ are conceptually affiliated with more than one broad domain (e.g., Originality and Constancy), reflecting the nuanced psychological constructs measured by the TPQ at the facet level. While confidence intervals for the standardised coefficients were calculated, the primary focus of this specific section of the analysis remained on the magnitude and theoretical context of the point estimates as part of this initial exploratory phase.

When matched up against the theoretical ideas about personality and interests these patterns make a lot of sense. For example, when it comes to Artistic interests, people with low levels of the altruistic and dutiful traits, combined with high levels of complexity, appear to thrive. That suggests that the artistic drive does best when it is free to run with ideas–without having to consider what is good for others or follow rules–and instead is left to focus on exploring abstract concepts. On the other hand, people who are into Conventional interests tend to do well in structured environments, not because they’re afraid of taking risks, but because they’re calm, patient, and just generally stable ( Kaufman et al., 2016).

**Figure 4.**
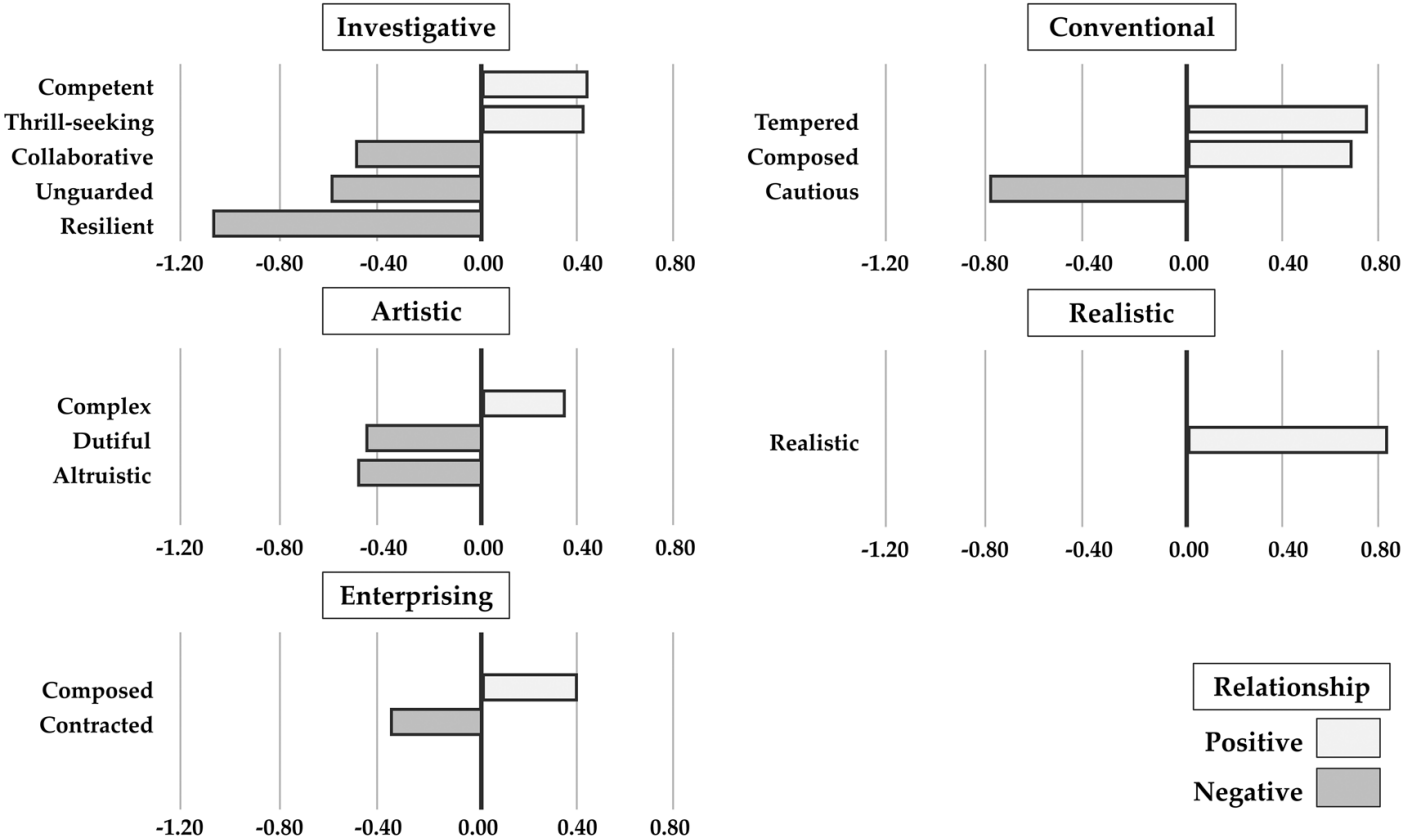
Visualised Personality-Interest Motivational Sequences (PIMS) for key RIASEC types. **Note.** Horisontal bar charts display the standardised regression coefficients ( *β*) of significant TPQ facet-level predictors for RIASEC types where a distinct personality signature was identified. Bars extending to the right of the central axis indicate facets that positively predict the interest; bars to the left indicate negative predictors. The length of the bar corresponds to the strength of the relationship. This visualisation reveals the distinct motivational sequences underpinning interest categories, such as the compensatory mechanism of high emotional stability offsetting low caution in Conventional types, and the complex blend of sensitivity to setback and intellectual drive in Investigative types. The Social (S) type is omitted due to the absence of any significant facet-level predictors, providing visual reinforcement of its inherent heterogeneity. Numerical values and statistical details are provided in Table 2.

Most notably, Investigative interests showed the most complex motivational sequence. This profile blended pronouncedly low Resilient–the strongest single predictor in the model–with low Sociability facets (Unguarded and Collaborative). This core of social detachment and sensitivity to setback was coupled with high Thrill-seeking and high Competent. This profile portrays the investigative innovator as an intellectually driven problem-solver, whose traits may ironically fuel a compensatory drive to master abstract challenges. Conversely, and importantly, no personality facets met the significance cut-off for predicting Social interests, a null finding visually underscored by its omission from Figure 4. This provides robust empirical support for critiques of within-type heterogeneity ( Roberts et al., 2006; Nye et al., 2017), suggesting the ‘Social’ category is a broad container for individuals with divergent motivational architectures.

### 3.4 Core traits in interest formation

Beyond predicting specific interest categories, a feature importance analysis (Mean Dropout Loss) was conducted to distinguish the personality facets most vital to the model’s overall predictive accuracy, as shown in Table 3. The top predictors were Dutiful, Relaxed, Composed, Resilient, and Unguarded. Consequently, this indicates that traits related to goal-directedness (conscientiousness), emotional stability (constancy), and trust (sociability) are fundamental, cross-cutting dimensions that powerfully influence the entire landscape of RIASEC vocational interest formation ( DeYoung, et al., 2016).

**Table 3.** Feature importance analysis for the PIMS model.

Rank	Personality facet	Mean dropout loss
1	Dutiful	576.44
2	Relaxed	531.10
3	Composed	525.08
4	Resilient	500.73
5	Unguarded	466.66
6	Sociable	452.27
7	Complex	424.67
8	Impervious	420.11
9	Tempered	393.34
10	Directed	381.26

**Note.** We used a logic similar to the “dropout” method in machine learning to establish which facet-level characteristics were most important to our model’s predictions. Similar to how dropout prevents a neural network from becoming unduly dependent on any one neuron by forcing it to learn robust, redundant representations, our feature importance analysis temporarily “dropped” information from each personality facet by permuting (rearranging a set of elements into a different order) its values. This process reveals the elements that the model has “learned” to depend on in order to produce precise forecasts. By measuring the drop in predictive accuracy when a facet is scrambled, we can distinguish traits that are fundamental to the motivational architecture of an interest category from those that are merely correlated or redundant. This approach ensures our identified Personality-Interest Motivational Sequences (PIMS) are based on solid, generalisable relationships between personality architecture and vocational interests, rather than on false correlations within the specific dataset. In terms of our table, the higher the dropout value, the more essential that personality facet is to the model’s ability to accurately predict vocational interests.

In summary, the results provide compelling, multi-faceted evidence for the PIMS framework. The model’s significant predictive accuracy, the identification of specific and interpretable facet-level sequences for most RIASEC types, and the strategically informative null finding for Social interests collectively demonstrate that vocational interests are systematic outcomes of an individual’s facet-level personality architecture.

## 4. Discussion

This study provides solid empirical support for a fundamental change in vocational psychology, demonstrating that vocational interests are best understood not as static alignments with environmental categories, but as dynamic outcomes of Personality-Interest Motivational Sequences (PIMS). The multinomial model’s ability to predict individuals’ primary RIASEC interest category with 60.0% accuracy using thirty personality facets substantiates this claim. This level of accuracy substantially exceeds chance-level prediction and improves upon the explanatory power of the moderate domain-level correlations (e.g., *r* ≈ .48, *R ^2^* = .23) that have long characterised the literature ( Barrick, et al., 2003; Larson, et al., 2002). Furthermore, it is important to note that the domain-level correlations we sought to improve upon are themselves substantively significant; the contribution of the PIMS model is not to dismiss these associations but to explain their underlying architecture and, in doing so, significantly enhance predictive precision and provide an explanatory narrative. The clear predictive accuracy of the PIMS model demonstrates that vocational interests are systematic and predictable outcomes of an individual’s facet-level personality architecture (cf. Larson, et al., 2002). However, arguably the most compelling validation of the PIMS framework comes not only from its predictive successes but also from a single, strategically informative failure: the model’s inability to find a unified personality signature for Social interests (S-CIP). This null finding provides robust empirical support for critiques of RIASEC’s heterogeneity ( Nye et al., 2017; Roberts et al., 2006) and strongly confirms the core PIMS proposition that vocational choices are made by individuals following distinct motivational sequences, not by assigning ‘types’. This inability to find a unified personality signature for Social interests resonate with earlier indications that broad interest categories can mask divergent underlying motivations. Costa, McCrae, and Holland (1984), for example, suggested that the correlation between extraversion and social/enterprising interests might be partly confounded by a general ‘breadth of interest’ factor. Our facet-level analysis circumvents this problem by examining specific trait combinations, thereby revealing that the apparent link to ‘Social’ interests is not a single drive but an aggregate of several distinct motivational pathways whose signals cancel each other out when analysed as a unified pattern.

This finding directly challenges the interpretation of meta-analytic results showing consistent, albeit modest, correlations between Social interests and Agreeableness ( *r* = .19) (TPQ; Sociability) and Extraversion ( *r* = .31) ( Larson et al., 2002). The PIMS model reveals that these domain-level correlations are likely an aggregate of divergent motivational sequences; for example, a nurturing ‘altruist’ higher in the Compassionate and Altruistic facets versus an influencing ‘leader’ higher in the Assertive and Sociable facets. Within the constraints of the RIASEC typology, these distinct psychological profiles generate conflicting statistical signals that prevent the identification of a single predictive pattern. Consequently, the modest domain-level correlations that have long characterised the literature ( Larson et al., 2002; Nauta, 2010) are likely an aggregate of these divergent signals, robustly confirming the core PIMS proposition that vocational choices are outcomes of individual trait configurations, not assignments to broad, homogeneous environmental types.

By operationalising interests as emergent outcomes rather than environmental matches and by modelling at the facet level, this study reveals the intricate trait interactions that earlier research has obscured. In so doing, the PIMS framework resolves the personality-interest contradiction by demonstrating that interests are not simply the sum of broad domains, but rather the product of their dynamic interplay ( McCrae & Costa, 1987). For instance, the Conventional profile shows a clear compensatory mechanism, where high emotional stability (Tempered, Composed) makes up for low levels of cautiousness ( De Fruyt & Mervielde, 1999), enabling individuals to flourish in orderly environments through patience and focus, as opposed to fear of error. Consequently, the Investigative profile, in turn, reveals a strong suppression effect, where low resilience intensifies a focus on intellectual mastery, perhaps to resolve intellectual discomfort or circumvent social challenges.

These subtle findings, which explain rather than merely describe interests, simultaneously serve as a critical lens on the fundamental limitations of the RIASEC model itself. The model’s difficulty in defining Social and Enterprising types, and its failure to identify a significant personality signature for Social interests altogether, are not shortcomings of the PIMS approach but are instead data-driven indications of RIASEC’s categorical constraints ( Nye et al., 2017; Tinsley, 2000). The finding that the ‘Social’ category served best as a statistical baseline, with no unique facet profile defining it positively, strongly reinforces the thesis that it is an artificially singular cluster which groups distinct psychosocial motivations (e.g., nurturing versus influencing) into a single category ( Hirschi & Jaensch, 2015). It is this inherent heterogeneity that causes the unique statistical signals of these subgroups to cancel out, preventing the identification of a single, unifying personality signature.

Furthermore, the unstable profile for the Realistic type, a result of its low prevalence in our university sample, also serves as a powerful conceptual illustration of the PIMS thesis. The classic ‘Realistic’ designation, centred on manual trades, agriculture, and direct tool manipulation ( Holland, 1997), may be a poor fit for a modern university population or society in general. Individuals with the underlying motivational drivers of ‘realism’ (e.g., high assertiveness, a focus on tangible outcomes, procedural competence) may now channel these traits into academically aligned pathways such as engineering, computer science, or applied research. The O*NET Profiler, bound to the RIASEC environmental typology, may misclassify these individuals into adjacent categories like Investigative or Enterprising, not because they lack realistic traits, but because the environmental descriptors of the ‘R’ type are culturally and contextually constrained. Consequently, this misalignment shows how the conflation of environmental tasks with psychological impulses within RIASEC artificially attenuates observable effect sizes by misaligning measurement with underlying motivational mechanisms ( Nauta, 2010).

Collectively, these findings underscore that vocational interests originate in personality, not in environments. The historical integration within RIASEC of the ‘where’ (environment) with the ‘why’ (motivation) has limited the field’s explanatory power and, consequently, its predictive power ( Berdie, 1944). This conflation is challenged by the empirical demonstration that personality and interests predict different outcomes ( De Fruyt & Mervielde, 1999), a finding which forces a conceptual reckoning with the typological model itself. Our findings at the facet-level extend this work by moving beyond how these constructs relate to explaining how specific configurations of personality traits dynamically give rise to the motivational outcomes captured by interest inventories. The strong performance of the PIMS model, even within this constrained taxonomic system, suggests that future research should use the PIMS framework to provide a personality-based explanation for the motivational constructs identified by inventories such as the MAPP, Career Anchors, and Work Values Inventory. By detecting the trait sequences that give rise to these motivations, we can move from a typological description of interests to a dynamic, explanatory model of career development.

### 4.1 Theoretical and practical implications

**Figure 5.**
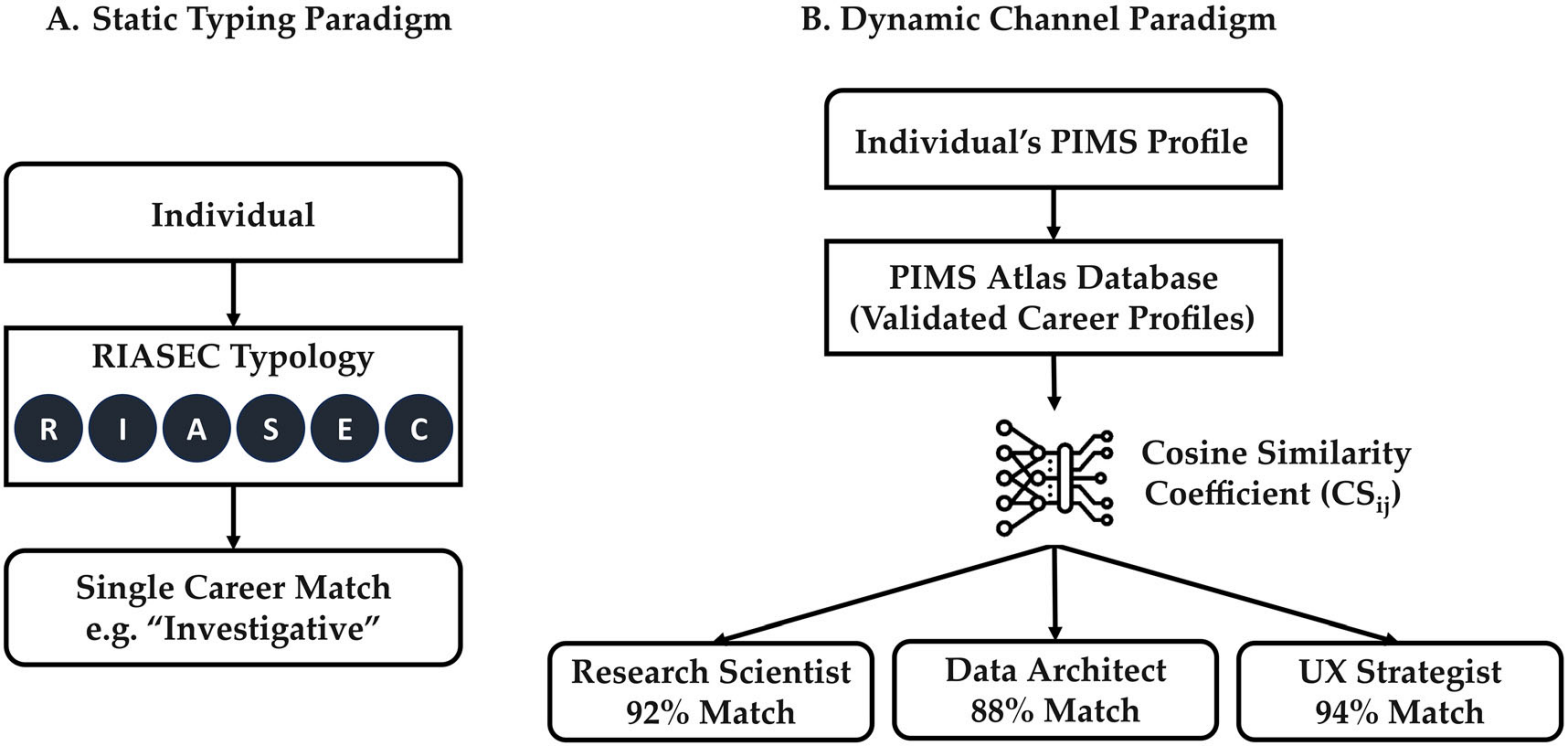
From static typing to dynamic pathwaying: A conceptual model for PIMS-based career navigation. **Note.** This schematic illustrates the paradigm shift enabled by the PIMS framework, moving beyond the constraints of static typologies (Panel A). The proposed future application (Panel B) conceptualises how an individual’s unique PIMS profile, derived from their facet-level personality architecture, can be algorithmically matched against a validated database of profiles from successful professionals (the PIMS Atlas). This process generates a dynamic and personalised set of potential career pathways, each with a quantified congruence score (Cosine Similarity Coefficient (CS _ij_)), rather than a single categorical assignment. Congruence scores are calculated using cosine similarity (CS), which measures the angular similarity between thirty personality facet vectors, with values ranging from -1 (perfect negative alignment) to 1 (perfect positive alignment). The congruence between Individual ( **i**) and Career ( **j**) is denoted as CS _ij_. This model positions PIMS as a unifying, explanatory framework capable of contextualising a wide array of motivational constructs (e.g., work values, career anchors) by tracing their origins to specific trait configurations.

The paradigm shift suggested by the PIMS framework holds significant theoretical and practical implications ( Borgen, 1999) for predicting meaningful educational and occupational outcomes ( Hirschi & Jaensch, 2015; Lechner et al., 2018). A substantial body of evidence confirms that vocational interests are key predictors of critical outcomes, including performance, persistence, and career success ( Rounds & Su, 2014). The PIMS framework provides a facet-level basis for moving beyond descriptive typologies and tapping into the generative mechanisms behind these outcomes. For example, Career Counselling PIMS profiles can show subtle alignments between traits and career aspirations, moving beyond broad personality domain-level matches. Specifically, an Investigative PIMS profile, characterised by low Resilience and Sociability coupled with high Thrill-seeking and Competence, can signal a strong propensity for R&D roles where intellectual risk-taking is valued, while also flagging a potential need for organisational support mechanisms to help the individual manage potential issues. Within Human Resources and Education, the PIMS framework offers a practical advance: by identifying the precise combinations of personality facets that predict success in a given role, it enables more nuanced selection and tailored development programmes. Looking ahead, the most significant contribution of PIMS may be its power to integrate the many existing models of motivation. As illustrated in Figure 5, the framework suggests that core motivational themes–such as the ‘Need for Autonomy’ or ‘Altruism’–are not independent constructs, but natural outcomes of specific, underlying personality trait configurations. This provides a unifying, personality-based explanation for what other inventories merely describe. Consequently, PIMS reimagines career guidance from a static exercise of matching traits to jobs into a dynamic, personalised process of charting an individual’s unique pathway. This would be operationalised through a PIMS Atlas: a dynamic, empirically derived database that catalogues the unique PIMS profiles of individuals who demonstrate high levels of success and satisfaction in specific occupational roles. A future application of this framework would involve a tailored list of career possibilities, each with a high degree of motivational alignment. This in turn provides each individual the opportunity to move beyond one-size-fits-all solutions, helping them create unique vocational paths that align with what motivates them personally.

**Figure 6.**
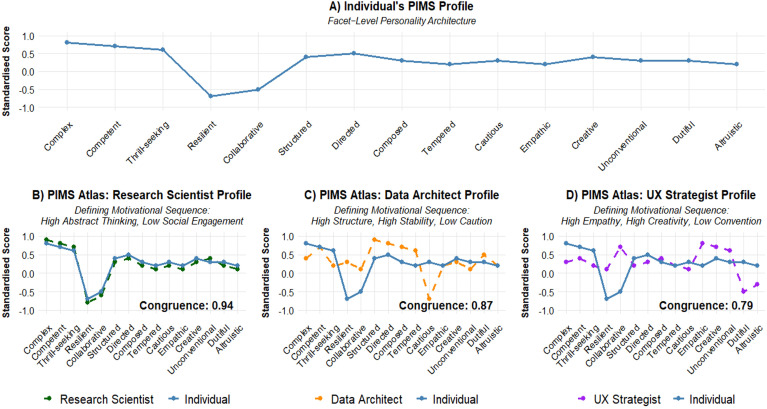
Practical application of the PIMS framework. **Note.** This framework represents a parallel trajectory matching of an individual’s facet-level PIMS profile (Panel A) against validated career profiles from the PIMS Atlas (Panels B-D). Congruence scores (CS _ij_), calculated via cosine similarity, quantify the motivational alignment for each career pathway. Higher congruence (e.g., 0.94 for Research Scientist) indicates a closer match between the individual’s personality-derived motivational sequence and the profile of success in that occupation, generating a ranked set of personalised recommendations rather than a single typological assignment.

In the following figure ( Figure 6) this conceptual framework demonstrates how individual PIMS profiles can be algorithmically matched against validated career profiles in the PIMS Atlas to generate personalised pathway recommendations.

In terms of Research, PIMS offers a solid framework for interpreting and contextualising the results of motivation-centric tools through the lens of personality. For example, a high score on the ‘Altruistic’ scale of the MAPP can be understood through the PIMS lens as likely stemming from facets high in Sociability (e.g., Compassionate, Altruistic) and low in certain aspects of Extraversion (e.g., Thrill-seeking).

The PIMS framework builds upon this integration in two crucial ways. First, it moves beyond descriptive correlation to offer genuine explanatory mechanisms. Second, it provides practitioners with actionable, facet-level narratives that translate abstract concepts into practical insights. Consequently, these narratives not only help individuals understand that their traits and interests are connected, but specifically how their unique personality profiles shape their vocational pathway. In a more practical sense, this awareness practically informs us how to bridge our theoretical understanding of what motivates us to follow specific vocational paths with tangible career development applications.

### 4.2 Limitations

Our study offers a compelling case for the PIMS framework, but there are some associated boundaries to consider. First, our findings are rooted in the experiences of a particular group: South African university students on the cusp of their careers. While this group is multicultural and highly relevant for studying career decision-making, we need to be cautious. The personality-interest patterns we identified might look different in people already established in their careers, in different age groups, or in cultural settings with dissimilar economic landscapes and opportunity structures. The next step is to see how well these motivational sequences translate beyond the academic context.

Second, while personality traits are often considered relatively stable foundations, our study’s design cannot capture how the interest side of the equation might fluctuate. We work from the premise that traits are fairly hard-wired, but vocational interests are not necessarily static endpoints–they can evolve, intensify, or fade through exposure, experience, and opportunity. Our model shows which trait configurations predict interest categories at a given moment, but it leaves open the question of how interests dynamically form and solidify around that personality core over time. A longitudinal view is needed to understand this maturation process.

Finally, there is an inherent irony in our methodological choice. To demonstrate the predictive power of our personality-based model, we used the RIASEC interest categories as our target–the very typology our framework seeks to move beyond. We are, in a sense, using the old map to prove the need for a new one. The most telling result here is the ‘null’ finding for Social interests, which does not indicate a failure of our model, but rather exposes the fundamental heterogeneity bundled into that single category. The true promise of PIMS lies in eventually replacing broad typological outcomes with more nuanced, personality-defined pathways, a task for the research that follows. These limitations, however, do not diminish the value of the PIMS framework; rather, they define the contours of the work still to be done.

### 4.3 Future research directions

Guided by these considerations, we propose several key avenues for future research. First, research should place the identified PIMS sequences in the context of motivational psychology. This entails doing joint validation studies that map PIMS-derived personality profiles onto scores from established inventories such as the MAPP, Career Anchors, and Work Values Inventory. To progress from identifying correlations to explicitly testing the proposed generative mechanisms, future studies should employ formal mediation analysis. For instance, longitudinal studies could assess whether specific facet-level traits (e.g., low Resilience and low Sociability) statistically mediate the development of Investigative interests over time, thereby providing causal pathway evidence for the PIMS sequences proposed in this study.

Second, a primary applied goal is the development of tools that translate theory into personalised guidance. This involves building the proposed PIMS Atlas: a dynamic database matching individual trait profiles to occupational pathways. The PIMS Atlas–in particular–presents a prime opportunity for applied machine learning where researchers could train and deploy complex recommender systems–using techniques such as collaborative filtering, gradient boosting, or neural networks–linking personality profiles to career outcomes. These algorithms would be uniquely suited to model the complex, high dimensional interactions between the thirty TPQ facets and diverse career outcomes, generating the personalised congruence scores (e.g., CS _ij_) conceptualised in Figure 5.

Finally, the inherent heterogeneity of broad interest categories must be dismantled empirically. Person-centred analytical techniques, such as latent profile analysis on facet-level data, should be used to identify data-driven subtypes within RIASEC categories. This approach would empirically test the proposition that, for example, the Social domain comprises distinct subgroups like a nurturing ‘Altruist’ and an influencing ‘Leader’, thereby resolving the enigma of the null finding for this type in the present study.

By pursuing this integrated agenda–from mechanistic validation and algorithmic application to contextual generalisation–the PIMS framework can evolve from a compelling proposition into the backbone of a more dynamic, personalised, and explanatory science of career development.

## 5. Conclusion

In conclusion, this study reformulates vocational interests as dynamic products of Personality-Interest Motivational Sequences (PIMS), demonstrating that career preferences crystallise through the confluence of specific personality facets rather than through a passive mirroring of environmental categories. The PIMS framework, therefore, addresses a foundational stalemate in vocational psychology. While research has definitively established that interests are powerful, trait-like predictors of critical outcomes such as performance, persistence, and career success ( Rounds & Su, 2014), the field has remained constrained by a paradigm that treats them as static categories for environmental matching. The PIMS framework bridges this gap by moving from description to explanation, providing a generative, mechanistic model for how these trait-like interests emerge from the dynamic interplay of facet-level personality traits. In essence, whereas Rounds and Su (2014) established the predictive power of interests, this study, by contrast, explains their very nature and origin within an individual’s personality architecture.

The robust predictive accuracy of the model provides compelling evidence that these sequences are systematic, non-random, and measurable. The identified sequences–such as the compensatory stability in Conventional profiles and the complex facet interplay in Investigative profiles–quantitatively define the very motivational pathways theorised by the PIMS framework, thereby moving the theory from proposition to empirical reality. While Holland’s (1997) RIASEC model provides a helpful general guide, its categories serve better as broad summaries of interest outcomes than as their primary source. The collective evidence demonstrates that vocational interests are emanations of deeper personality systems; consequently, empirically derived trait configurations predict an individual’s preferences more precisely than broad typological labels can. The PIMS perspective recasts RIASEC types as outcomes of personality dynamics rather than fixed boxes for classification, which, in turn, directly accounts for the heterogeneity within types. Broad categories like ‘Social’ are thus revealed not as single entities, but as common outcomes arising from several distinct underlying personality configurations.

Ultimately, the PIMS model resolves a century-old personality-environment conflation and provides a parsimonious explanation for why prior research plateaued at the modest correlations documented in reviews of the typological approach. More important, it provides a plausible explanation as to why existing research is stuck measuring the reflections of traits filtered through environmental categories, rather than the traits themselves. Consequently, the PIMS framework provides the foundation for transforming career guidance from static matching–categorising individuals into predefined types for alignment with occupational lists–into personality-informed pathwaying, which traces vocational preferences to their source in specific, facet level motivational sequences. It thus empowers individuals to understand how their deepest traits shape their professional narratives and enable them to build a vocational life that is not merely a fit, but an authentic expression of self.

## Ethics and consent

This study was approved by The IIE Ethics Committee, Independent Institute of Education, Emeris (R.1602223 [REC]). Written informed consent for the primary questionnaires was obtained via an email outlining the study’s purpose, anonymity, voluntary nature, and a statement highlighting that navigating the included link and submitting the assessments would constitute informed consent – *“Should this research be of interest to you, please complete the survey at the link below. By completing the questionnaire, you are automatically giving consent to the researchers”.*

## Authors note

Gary Clifford Townsend [ https://orcid.org/0009-0001-0300-1522] is a Research Associate at the Independent Institute of Education, Emeris, Faculty of Humanities.

Portia Webb [ https://orcid.org/0009-0003-7202-7026] is the Head of Commerce, Independent Institute of Education, Emeris, Faculty of Commerce.

Correspondence concerning this article should be addressed to Gary Clifford Townsend.

Email: gary.townsend43@gmail.com or gary@skillworxafrica.com

## Data Availability

**Repository name:** Underlying Data - Decoding Decisions: The Predictive Power of PIMS. https://doi.org/10.6084/m9.figshare.c.8271115. [ Townsend, G. C., & Web, P. (2023)]. *Underlying Data - Decoding Decisions: The Predictive Power of PIMS* [Data set]. Figshare. https://doi.org/10.6084/m9.figshare.c.8271115 The project contains the following underlying data: PIMS TPQ & RIASEC Scores (504). (formatted [scores reversed] raw Likert scores). **Repository name:** Extended Data - Decoding Decisions: The Predictive Power of PIMS. https://doi.org/10.6084/m9.figshare.c.8271796. [ Townsend, G. C., & Web, P. (2026)]. *Extended Data - Decoding Decisions: The Predictive Power of PIMS* [Research instruments and supplemental files]. Figshare. https://doi.org/10.6084/m9.figshare.c.8271796 This project contains the following extended data: PIMS Variables & Descriptives. (TPQ and RIASEC variables and their descriptions/definitions) PIMS Abbreviations. (Abbreviations descriptions used in the manuscript) PIMS Visual Abstract. (A visual representation of the entire PIMS framework and process) PIMS Research Invitation to Participate. (The participant invitation to participate email) PIMS Questionnaire Items **.** (An excel file with both the TPQ and RIASEC assessment items and instructions). The data and research instruments supporting this study are openly available in Figshare. Both datasets are used under the terms of the Creative Commons Attribution 4.0 International (CC BY 4.0) license. All software used for analysis are open source and readily available online. These can be accessed here: https://www.jamovi.org/ and https://www.r-project.org/. Not applicable.
