# Supplementary material for: Decoding Decisions: Personality-Interest Motivational Sequences as Predictors of Career Paths
Source: F1000Res. 2026 Jun 24;15:220. Originally published 2026 Feb 10. [Version 3] doi: 10.12688/f1000research.176767.3 (PMC13324359; doi:10.12688/f1000research.176767.3)
Supplement: Peer review history

# Reviewer report: Reviewer response for version 3

**DOI:** 10.5256/f1000research.204034.r496335

**Published:** 2026-07-01

**Authors:** Melinde Coetzee

**Affiliations:** 1 University of South Africa, Pretoria, Gauteng, South Africa

The authors addressed my concerns satisfactorily. Their revisions enhanced the quality of the paper.

Is the work clearly and accurately presented and does it cite the current literature?

Yes

If applicable, is the statistical analysis and its interpretation appropriate?

Yes

Are all the source data underlying the results available to ensure full reproducibility?

No source data required

Is the study design appropriate and is the work technically sound?

Yes

Are the conclusions drawn adequately supported by the results?

Yes

Are sufficient details of methods and analysis provided to allow replication by others?

Yes

Reviewer Expertise:

Career and Vocational Psychology

I confirm that I have read this submission and believe that I have an appropriate level of expertise to confirm that it is of an acceptable scientific standard.

# Reviewer report: Reviewer response for version 3

**DOI:** 10.5256/f1000research.204034.r496334

**Published:** 2026-06-26

**Authors:** John A. Johnson

**Affiliations:** 1 The Pennsylvania State University, University Park, Pennsylvania, USA

The article is even stronger now. I especially appreciated the explicit definitions of personality and vocational interests at the outset of the paper.

Is the work clearly and accurately presented and does it cite the current literature?

Partly

If applicable, is the statistical analysis and its interpretation appropriate?

Yes

Are all the source data underlying the results available to ensure full reproducibility?

Partly

Is the study design appropriate and is the work technically sound?

Yes

Are the conclusions drawn adequately supported by the results?

Partly

Are sufficient details of methods and analysis provided to allow replication by others?

Yes

Reviewer Expertise:

Personality and vocational theory and assessment; evolutionary psychology.

I confirm that I have read this submission and believe that I have an appropriate level of expertise to confirm that it is of an acceptable scientific standard.

# Reviewer report: Reviewer response for version 2

**DOI:** 10.5256/f1000research.202255.r492130

**Published:** 2026-06-13

**Authors:** Melinde Coetzee

**Affiliations:** 1 University of South Africa, Pretoria, Gauteng, South Africa

Overall, a valuable and novel study that contributes important new insights to the vocational psychology field. The paper is well-written and provides evidence of excellence in research scholarship and scientific rigor.

 Background: second sentence “This plateau…..theory, treat….”: the sentence needs rephrasing as the meaning is not clear

 Introduction: the authors provide a scientifically rigorous review of the research problem and a convincing justification for their study. The arguments are presented in a logical, systematic manner that elucidates the research problem and the implications of the research gaps - if not addressed- for the field of vocational psychology. All arguments are substantiated by means of both classical and contemporary research literature.

 However, they need to also explain the terms personality (including personality trait-like dispositions) and vocational interests as relevant to the present study. Currently, the meaning of the key constructs is not clear and constructs are generally ill-defined. Construct meanings (as relevant to the study) are important to better understand the authors’ later argument that their PIMS framework models generative psychological mechanisms.

 The introduction needs to include a clear statement of the objective of the study and the value that will be added to the field of vocational psychology. The objective statement will add better perspective on the sections that follow.

 Point 1.2: 1 ^st^ paragraph- the authors refer to the PIMS framework.. claiming that the PIMS framework extends the cited work in the previous argument- however, it is not clear what they mean with the abbreviation of PIMS framework. Authors should provide full terminology of PIMS with the abbreviation in brackets (1 ^st^ time reference to PIMS framework). The notions of generative mechanisms and motivational sequences  within the PIMS framework should also be explained.

 Point 1.2: 2nd paragraph – the authors refer to the PIMS framework as a causative framework- in their revised comments- they stated that they replaced the term causative with explanatory- the sentence/argument in the 2 ^nd^ paragraph of Point 1.2 needs to be revised accordingly. The term explanatory is more appropriate than the term causative.

 Point 1.2: 2nd paragraph – the authors need to explain the constructs that the TPQ measures and why the TPQ is of relevance/appropriate to achieving the objective of the study.  How do the TPQ personality dimensions relate to trait theory and facet-level personality traits, including motivational themes? This explanation is essential to make better sense of the arguments presented in point 1.4.

 Point 1.3: The authors provide a convincing (scientifically justified) argument pertaining to the novelty and utility of the PIMS framework in vocational psychology.

 Figure 1: The authors need to explain whether the personality dimensions outlined in the figure align with the TPQ measure (their literature review does not address these dimensions). They also need to explain/justify to what extent these personality dimensions relate to the notion of motivational sequences of the RIASEC vocational interests.

 Point 1.4:  A reference should be cited for MAPP.

 Point 2: Methods- the authors state the study objective (primary aim) which should be provided in the Introduction section as part of the solution for addressing the research problem.

 In the method section the authors state their study was a quantitative, cross-sectional design. In the procedures section- they refer to obtaining explicit consent for an optional longitudinal study- more clarification is needed.

 Participants: the term years should be added to the ages, eg 21 and 25 years

 Measures: Just a note- protocol is to integrate the psychometric properties with the description of the measures- not as separated headings.

 Statistical procedures are aligned with the primary aim/objective and research questions of the study.

 The results are appropriately reported and provide evidence of scientific rigour.

 Point 4: Discussion: The authors applied scientific rigor in elucidating the meaning of the statistical/empirical findings and to provide evidence for the utility of the PIMS framework in vocational psychology in the theoretical and practical implications section. The practical examples add new insights and enrich the quality and value of the study findings.

 The authors critically evaluated the limitations and provided meaningful recommendations for future research.

 The conclusion can be more concise – currently a lot of repetitive information.

Is the work clearly and accurately presented and does it cite the current literature?

Yes

If applicable, is the statistical analysis and its interpretation appropriate?

Yes

Are all the source data underlying the results available to ensure full reproducibility?

No source data required

Is the study design appropriate and is the work technically sound?

Yes

Are the conclusions drawn adequately supported by the results?

Yes

Are sufficient details of methods and analysis provided to allow replication by others?

Yes

Reviewer Expertise:

Career and Vocational Psychology

I confirm that I have read this submission and believe that I have an appropriate level of expertise to confirm that it is of an acceptable scientific standard, however I have significant reservations, as outlined above.

# Response

**Published:** 2026-06-14

**Authors:** Gary Clifford Townsend

**Affiliations:** Humanities, Independent Institute of Education, Emeris, Port Elizabeth, Eastern Cape, South Africa

Thanks, Prof. Coetzee, for your thoughtful and constructive review of our manuscript. We appreciate your recognition of the study’s value, scientific rigour, and contribution to vocational psychology. Below we provide a detailed, itemised response to each of your points, noting where we can make strategic revisions to strengthen our thesis. We specifically provide the detailed markup notes here for your reference and will reload the refined version incorporating all your feedback shortly hereafter.

**Responses to Review Guidance**

**Response to Background Sentence Rephrasing**

**Point 1:** We note your observation that the second sentence of the Background section requires rephrasing.

**Our Response:** 

 We agree that the interruption of the citation and the ambiguous referent for “they” impede readability. We will rephrase to clarify the meaning.

**Proposed Revision** **(Background section):**

**Original:** *“This plateau occurs because dominant models, such as Holland’s RIASEC (Realistic, Investigative, Artistic, Social, Enterprising, Conventional) theory, treat interests as fixed matches to environments, rather than as dynamic outcomes of an individual’s psychology.”*

**Revised:** *“This plateau stems from a fundamental limitation in dominant models (e.g., Holland’s RIASEC theory): they conceptualise interests as static matches between individuals and environmental categories, rather than as dynamic outcomes emerging from an individual’s underlying psychological architecture.”*

**Response to Construct Definitions (Personality and Vocational Interests)**

**Point 2:** We note your observation that the manuscript would be served by explicit definitions of personality and vocational interests as relevant to the study, which would help readers understand the PIMS framework’s explanatory claims.

**Our Response:** 

 This is a helpful observation. While these constructs are discussed implicitly throughout, a dedicated definition early in the Introduction will strengthen the manuscript’s foundation. We will add a new paragraph after the opening of the Introduction.

**Proposed Revision** **(Introduction, new paragraph after first paragraph):**

 “Before proceeding, it is useful to clarify how this study conceptualises its two central constructs. Personality refers to the relatively stable, enduring patterns of thoughts, feelings, and behaviours that distinguish individuals. Within the Five‑Factor Model framework adopted here, personality comprises broad domains (e.g., Extraversion, Conscientiousness) that can be further disaggregated into facet‑level traits (e.g., Sociable, Dutiful, Resilient). These facet‑level traits represent the granular, motivational building blocks of personality architecture. Vocational interests, by contrast, refer to individuals’ trait‑like preferences for specific activities, work environments, or occupational domains (Rounds & Su, 2014). They are relatively stable over time and predict critical career outcomes including job choice, performance, and persistence. Critically, while personality and vocational interests are related (meta‑analytic correlations around *r* = .48), they are empirically distinct constructs. The theoretical gap this study addresses is not whether they relate, but how and why - specifically, what generative psychological mechanisms produce these interests from an individual’s personality architecture.”

**Response to Study Objective and Value Statement**

**Point 3:** Here you suggest that the Introduction would benefit from a clear statement of the study’s objective and the value it adds to vocational psychology, placed before the research questions.

**Our Response:** 

 We agree. The research questions appear at the end of the Introduction, but an upfront objective statement will better orient readers. We will add this after the construct definitions.

**Proposed Revision** **(Introduction, positioned as described above):**

*“The present study aims to move the field beyond the persistent ‘correlation plateau’ (r ≈ .48) by introducing and empirically testing the Personality* *‑* *Interest Motivational Sequences (PIMS) framework. Rather than merely describing that personality and interests correlate, we seek to explain why and how specific patterns of facet* *-* *level personality traits generate vocational interests. The value added to vocational psychology is threefold: (1) methodologically decoupling personality from environmental typologies to enable clearer inference about interest formation; (2) providing a generative, mechanistic account of interest emergence that current descriptive models lack; and (3) offering an applied framework for dynamic, personalised career pathwaying that moves beyond static RIASEC matching.*”

**Response to PIMS Abbreviation – First Use in Introduction**

**Point 4:** You correctly note that the abbreviation PIMS is used in the Introduction before the full term has been provided (though it does appear in the Abstract).

**Our Response:** 

 Agreed. While the Abstract defines PIMS, the first use in the Introduction body should also include the full term for reader convenience. We will make this adjustment.

**Proposed Revision** **(Section 1.2, first paragraph):**

**Change:** *“The PIMS framework extends this work by modelling the combined, interactive effects...”*

**To:** *“The Personality‑Interest Motivational Sequences (PIMS)...”*

**Response to Explanation of Generative Mechanisms and Motivational Sequences**

**Point 5:** We note that you recommend explaining what is meant by “generative mechanisms” and “motivational sequences” within the PIMS framework.

**Our Response:** 

 We agree that these terms deserve explicit definition at first mention. We will add a concise explanation.

**Proposed Revision** **(Section 1.2, immediately after introducing the full PIMS term):**

*“By ‘generative mechanisms,’ we refer to the psychological processes through which specific configurations of facet‑level personality traits produce vocational interests as emergent outcomes - rather than interests merely correlating with traits. By ‘motivational sequences,’ we mean the patterned combinations of relative strengths and weaknesses across multiple facets (e.g., low Resilience AND low Sociability AND high Thrill‑seeking) that collectively predispose an individual toward particular interest domains. These sequences are ‘motivational’ because they describe the directional and energetic properties of behaviour that underlie interest formation.”*

**Response to Causative vs. Explanatory Framework**

**Point 6:** You note that we stated we had replaced the term “causative” with “explanatory” in the second paragraph of Point 1.2.

**Our Response:** 

 You are correct. The sentence in section 1.2, second paragraph, line 4 should read: “...thereby moving from modelling correlation (cf. Etzel et al., 2025) to proposing an explanatory framework.”

**Proposed Revision** **(Section 1.2, Causative to Explanatory Framework):**

 Section 1.2, second paragraph, line 4 now reads: “...thereby moving from modelling correlation (cf. Etzel et al., 2025) to proposing an explanatory framework.”

**Response to Explanation of the TPQ and Its Appropriateness**

**Point 7:** We also note your suggestion that the Introduction should explain what the TPQ measures and why it is appropriate for testing the PIMS framework, particularly how its dimensions relate to trait theory and facet‑level measurement.

**Our Response:** 

 This is a valuable suggestion. Although the TPQ is described in the Methods section, a brief introduction to its facet‑level structure and Rasch validation will help readers understand why it is uniquely suited to this study.

**Proposed Revision** **(added at the end of Section 1.2 or as a new short paragraph):**

*“To operationalise this framework, we require a personality measure capable of assessing personality at the facet level - the granular units hypothesised to combine into motivational sequences. The Townsend Personality Questionnaire (TPQ; Townsend, 2017) is well‑suited for this purpose. Unlike many Big Five measures that report only domain‑level scores (e.g., global ‘Openness’), the TPQ assesses 30 distinct facet‑level traits - six facets per domain - each measuring a specific, psychologically meaningful behavioural tendency (e.g., ‘Thrill‑seeking’ under Extraversion; ‘Resilient’ under Constancy [inverse Neuroticism]; ‘Complex’ under Originality [Openness]). Critically, the TPQ was developed and validated using Rasch measurement, ensuring that scores meet the linear, interval‑scale properties required for the multinomial regression modelling central to our analytic strategy. These 30 facets serve as the independent variables in our model, allowing us to identify which specific combinations - or ‘motivational sequences’ - predict each vocational interest category.”*

**Response to Figure 1 Alignment with TPQ and Justification**

**Point 8:** We also note that you ask whether the personality dimensions in Figure 1 align with the TPQ measure and requests justification for how these dimensions relate to motivational sequences and RIASEC interests.

**Our Response:** 

 This is a fair point. Figure 1 lists the five TPQ dimensions and their facets, but the figure caption and surrounding text do not explicitly justify the relationship between these dimensions and RIASEC categories. We will revise the figure caption for additional clarity.

**Proposed Revision:**

**Figure 1 Caption (revised):** *“Figure 1. The Personality‑Interest Motivational Sequences (PIMS) Model. Note: This conceptual model illustrates how facet‑level personality traits from the Townsend Personality Questionnaire (TPQ) combine into motivational sequences. The TPQ measures five broad personality dimensions (Extraversion, Sociability, Constancy, Conscientiousness, Originality), each comprising six specific facets (e.g., Thrill‑seeking, Resilient, Complex). These facet‑level configurations are hypothesised to predispose individuals toward specific RIASEC vocational interest categories (Realistic, Investigative, Artistic, Social, Enterprising, Conventional), which are defined as environmental preferences rather than personality equivalents. The framework represents a paradigm shift from static typological matching to a dynamic model of personality‑driven pathwaying.”*

**Response to Missing MAPP Reference**

**Point 9:** Here you note that a reference should be cited for the MAPP (Motivational Appraisal of Personal Potential) mentioned in Section 1.4.

**Our Response:** 

 An oversight on our part. We will add the appropriate citation.

**Proposed Revision** **(Section 1.4):**

**Original:** *“...established motivational inventories such as the Motivational Appraisal of Personal Potential (MAPP), Schein’s (1990) Career Anchors, or Super’s (1970) Work Values Inventory.”*

**Revised:** *“...established motivational inventories such as the Motivational Appraisal of Personal Potential (MAPP; Assessment.com, 2026), Schein’s (1990) Career Anchors, or Super’s (1970) Work Values Inventory.”*

**Response to Study Objective Placement (Methods vs. Introduction)**

**Point 10:** You further observe that the primary aim statement appears in the Methods section and should be relocated to the Introduction.

**Our Response:** 

 We agree. It sets up the research questions more effectively here. We will move it accordingly.

**Proposed Revision:**

**Remove from Methods (Section 2):** Delete the sentence *“The primary aim was to investigate whether facet‑level personality traits could predict primary vocational interest categories, thereby operationalising the proposed Personality‑Interest Motivational Sequences (PIMS) framework.”*

**Add to Introduction:** *“To this end, the primary aim of this study was to investigate whether facet* *‑* *level personality traits could predict primary vocational interest categories, thereby operationalising the proposed Personality‑Interest Motivational Sequences (PIMS) framework.”*

**Response to Longitudinal Consent Clarification**

**Point 11:** You also requested clarification regarding the optional longitudinal component mentioned in the procedures section.

**Our Response:** 

 This is a fair point. The current text mentions explicit consent for a longitudinal component but does not specify what that entails. We will expand the description.

**Proposed Revision** **(Section 2.4, Procedure):**

**Original:** *“Conversely, explicit consent for the optional longitudinal component was obtained via a dedicated opt‑in field where participants voluntarily provided their email addresses for future contact.”*

**Revised:** *“Conversely, explicit consent for the optional longitudinal component was obtained via a dedicated opt‑in field where participants voluntarily provided their email addresses for future contact. This longitudinal component involves follow-up assessments post-graduation to track actual career choices and occupational outcomes, enabling future tests of the PIMS framework’s predictive validity for real-world career pathways.”*

**Response to Age Formatting (“years”)**

**Point 12:** You also note that the term “years” should be added to the age ranges (e.g., “21 and 25 years”).

**Our Response:** 

 A formatting correction we agree with. We will make the change.

**Proposed Revision** **(Section 2.1, Participants):**

**Original:** *“The majority were aged between 21 and 25 (70.9%), with 23.1% in the 14‑20 range...”*

**Revised:** *“The majority were aged between 21 and 25 years (70.9%), followed by 23.1% in the 14-20 years range, 4.8% between 26 and 30 years, and only 1.2% older than 30 years.”*

**Response to Psychometric Properties Integration (Stylistic Note)**

**Point 13:** Here you note that protocol typically integrates psychometric properties with the description of measures rather than using separate headings.

**Our Response:** 

 We appreciate your attention to these formatting conventions. We will integrate the psychometric properties directly into the measure descriptions (now under 2.2.1 and 2.2.2).

**Proposed Revision** **(Section 2.2.1 and 2.2.2, Measures):**

 The separate section 2.3 and its subheadings will be removed. No content will change.

**Response to Conciseness of the Conclusion**

**Point 14:** We note your suggestion that the conclusion could be more concise.

**Our Response:** 

 The conclusion does reiterate points already made in the Discussion. We will condense it while preserving the key contributions.

**Proposed Revision** **(Section 5, Conclusion – condensed version):**

*“In conclusion, this study reformulates vocational interests as dynamic products of Personality‑Interest Motivational Sequences (PIMS), demonstrating that career preferences crystallise through the confluence of specific personality facets rather than through passive mirroring of environmental categories. The PIMS framework addresses a foundational stalemate in vocational psychology by moving from description to explanation - providing a generative, mechanistic model for how trait‑like interests emerge from facet‑level personality architecture. The robust predictive accuracy (60.0% vs. 16.7% chance) provides compelling evidence that these sequences are systematic and measurable. Importantly, the null finding for Social interests exposes the heterogeneity masked by broad RIASEC categories, confirming that ‘Social’ is not a single personality signature but an aggregate of distinct motivational pathways. Ultimately, the PIMS model resolves a century‑old personality‑environment conflation and provides a parsimonious explanation for why prior research plateaued at modest correlations. It thus transforms career guidance from static typological matching into personality‑informed pathwaying - empowering individuals to understand how their deepest traits shape their professional narratives and to build vocational lives that are not merely fits, but authentic expressions of self.”*

# Reviewer report: Reviewer response for version 2

**DOI:** 10.5256/f1000research.202255.r490000

**Published:** 2026-06-11

**Authors:** John A. Johnson

**Affiliations:** 1 The Pennsylvania State University, University Park, Pennsylvania, USA

The authors have responded superbly to my original review, revising their manuscript accordingly. I approve of the manuscript as it now stands.

Is the work clearly and accurately presented and does it cite the current literature?

Partly

If applicable, is the statistical analysis and its interpretation appropriate?

Yes

Are all the source data underlying the results available to ensure full reproducibility?

Partly

Is the study design appropriate and is the work technically sound?

Yes

Are the conclusions drawn adequately supported by the results?

Partly

Are sufficient details of methods and analysis provided to allow replication by others?

Yes

Reviewer Expertise:

Personality and vocational theory and assessment; evolutionary psychology.

I confirm that I have read this submission and believe that I have an appropriate level of expertise to confirm that it is of an acceptable scientific standard.

# Reviewer report: Reviewer response for version 1

**DOI:** 10.5256/f1000research.194864.r482321

**Published:** 2026-05-11

**Authors:** John A. Johnson

**Affiliations:** 1 The Pennsylvania State University, University Park, Pennsylvania, USA

Review of Decoding Decisions: Personality-Interest Motivational Sequences as Predictors of Career Paths

 The current study seeks to surpass an alleged plateau of the correlation between personality traits and vocational interests of *r* ≈ .48 by employing Big-Five facet scales rather than domain scales as predictors of Holland personality types. The researchers also introduce a conceptual framework they call PIMS (Personality-Interest Motivational Sequences) that regards facet scores as reflecting dynamic motivational causes that generate vocational interests. The model furthermore reconceptualizes vocational interests as emergent outcomes of the interaction of facet-level motives rather than static attraction to the features of particular work environments.

 I would like to make a couple of preliminary points before addressing the details of the research study. First, given that personality traits and vocational interests are different (albeit related) constructs, there is no need to show correlations between traits and interests higher than r ≈ .48. After all, *r* ≈ .48 is what we generally find between personality traits assessed by self-report and the same traits assessed by pooled ratings from knowledgeable acquaintances. Second, comparing simple Pearson *r*s between single domain scales and interest scales to a multiple *R* between a set of facet scales and interest scales is not a fair comparison. A multiple *R* is bound to be larger than a simple Pearson *r*.

 That said, it would be nice to see trait-interest relationships to be stronger than what is found in the literature. The *r* ≈ .48 figure is actually generous as a purported maximum, as correlations of this magnitude are generally found only between Artistic vocational interests and Openness to Experience. Enterprising (and sometimes Social) interests tend to correlate with Extraversion in the high .30s at best, while Conventional interests sometimes correlate with Conscientiousness around .30 and sometimes in the .20s. Realistic and Investigative interests seem to have a ceiling of .20 or so, with an inconsistent pattern with Big-Five domain scales.

 I agree with the authors that examining relationships between vocational interests and facet-level Big-Five scores is a worthwhile endeavor. The question is whether such research has already been conducted. Despite an otherwise excellent review of the literature on the relationship between the five major personality factors and vocational interests, the current authors have apparently missed at least two published studies on this topic:

 [Reference 1]The five-factor model of personality and Holland's RIASEC interest types. *Personality and Individual Differences*, *23*(1), 87-103.

 and a replication and extension study,

 [Reference 2] Personality facets and RIASEC interests: An integrated model. *Journal of Vocational Behavior, 75*(3), 346–359. 

 I am not sure how the current authors missed these studies, because they do cite a De Fruyt and Mervielde (1999) article that cites the 1997 article. The authors give the mistaken impression that their research is the first to examine relationships between Big-Five facets and vocational interests. This needs to be corrected. Obviously the current authors need to compare their results to the previously published work on this topic.

 When the authors do compare their results to other studies, it would be helpful if they used the same metric. I do like their Precision, Recall, and F1 indices of correct prediction, but they should also report regression results used by other researchers. This is particularly important because they begin the article with the alleged ceiling of *r* ≈ .48 (a correlation), but it is not easy to see if they broke that ceiling when they report predictive accuracy. (Standardized beta regression coefficients are reported, but not overall *R*s.)

 Another issue that the authors might address is their acknowledgment of the irony in trying to predict the six discrete Holland type categories with continuous personality facets. Although Holland does refer to six personality-vocational "types," he and everyone who works with the Holland instruments is well aware that assessing either people or jobs in terms of discrete categorical types is insufficient. The Holland types might be thought of as "ideal types" in the sense of Max Weber, as useful theoretical fictions. But when it comes to assessment, RIASEC scores are continuous, meaning that people (and jobs) resemble the six types by degrees rather than an either/or categorization. Furthermore, Holland practitioners use 3-letter codes corresponding to the most prominent, second most prominent, and third most prominent feature of a person's personality or a work environment. A person (or work environment) with an AIR profile differs considerably a person or environment with an AES profile. If we want to use personality measures to guide people into the best possible job match, we need to consider at least their three highest scores (if not all six) for optimal placement.

 Finally, I should note that not everyone is going to agree that personality facet scores represent dynamic motives that cause vocational interests to emerge just because the authors declare that this is the case. I happen to believe that personality facets have motivational aspects to them, but other personality researchers regard facets as behavioral summaries or self-presentations. Furthermore, the claim that vocational interests are emergent consequences of a sequence of personality facets is more of a metaphysical assumption similar to the whole being more than the sum of its parts, rather than a statement of fact. Again, I might believe this, but others will not. Perhaps it is best if the authors emphasize that this aspect of PIMS is purely hypothetical and needs further evidence. Or, they might back down on the claim of emergence after reviewing the debate between Jack Block and Mendelsohn, Weiss, and Feimer on whether combinations of personality traits result in emergent properties beyond the traits considered separately.

 I appreciate the detailed documentation of the measures used in the study, which are surely sufficient for someone to replicate the study. I marked this aspect as only "partial" because the raw data itself does not seem to be available.

Is the work clearly and accurately presented and does it cite the current literature?

Partly

If applicable, is the statistical analysis and its interpretation appropriate?

Yes

Are all the source data underlying the results available to ensure full reproducibility?

Partly

Is the study design appropriate and is the work technically sound?

Yes

Are the conclusions drawn adequately supported by the results?

Partly

Are sufficient details of methods and analysis provided to allow replication by others?

Yes

Reviewer Expertise:

Personality and vocational theory and assessment; evolutionary psychology.

I confirm that I have read this submission and believe that I have an appropriate level of expertise to confirm that it is of an acceptable scientific standard, however I have significant reservations, as outlined above.

## References

[1] : The five-factor model of personality and Holland's RIASEC interest types. *Personality and Individual Differences* .1997;23(1) : 10.1016/S0191-8869(97)00004-4 87-103 10.1016/S0191-8869(97)00004-4

[2] : Personality facets and RIASEC interests: An integrated model. *Journal of Vocational Behavior* .2009;75(3) : 10.1016/j.jvb.2009.05.004 346-359 10.1016/j.jvb.2009.05.004

# Response

**Published:** 2026-05-12

**Authors:** Gary Clifford Townsend

**Affiliations:** Humanities, Independent Institute of Education, Emeris, Port Elizabeth, Eastern Cape, South Africa

Thank you Prof. Johnson for such a detailed and constructive review. Your feedback has materially improved the manuscript. We appreciate your insightful identification of areas where our manuscript would benefit from clarification, additional citations, and a more nuanced presentation of our claims. We will address each point in turn, and we have marked up our manuscript for specific revisions to strengthen our thesis and accommodate your feedback.

 We specifically provide the detailed markup notes here for your reference and will reload the refined version incorporating all your feedback shortly hereafter.

**Responses to Review Guidance**

**POINT 1: **“The plateau of *r* ≈ .48 is not a problem; it is what we expect for different constructs”

 We note your observation that *r* ≈ .48 is not a “plateau” needing to be surpassed - that this is roughly the level of self-other agreement for personality traits, and that personality and interests are different constructs.

**Our Response:** We agree that personality and vocational interests are distinct constructs, and we are not seeking to “break” the correlation in the sense of achieving a higher bivariate coefficient. In our assessment, the plateau is a *theoretical problem*, not a statistical one. It reflects the field’s inability to move beyond describing static associations to explaining their source. This is precisely why we developed the PIMS framework: not to increase the magnitude of the correlation, but to provide a generative, mechanistic explanation for *why* these relationships exist.

**Our Revision:**

**Abstract:** We will change *“exceeding the explanatory power of domain-level correlations”* to *“providing a generative explanation for the domain-level correlations that have long characterised the literature”*.

**Introduction:** Here we will add a sentence explicitly stating that our goal is not to achieve higher correlations but to explain their underlying architecture – *“We seek not to surpass the modest domain* *‑* *level correlations (r* *≈* *.48) that have long characterised the literature, but to explain the generative mechanisms that produce these associations.”*

**POINT 2: **“Comparing simple Pearson r to multiple R is not a fair comparison”

 You correctly note that a multiple regression R between facet scales and an interest scale is bound to be larger than a simple Pearson *r* between a single domain scale and an interest scale.

**Our Response:** This is an accurate observation. Our paper, however, does not directly compare domain-level *r* to the PIMS model’s multiple R. The 60% accuracy we report is a *classification accuracy* - the percentage of correctly predicted primary RIASEC categories from multinomial logistic regression - not a multiple correlation. This is a different metric. We do note that the model’s accuracy *“improves upon the explanatory power of the moderate domain-level correlations,”* but we are careful to frame this as an improvement in *explanation* (moving from description to mechanism) rather than a direct statistical comparison.

**Our Revision:**

**Results:** Clarify that the 60% accuracy is calculated on a held-out test set and represents correct classification of six categories, a different metric from domain-level *r* – *“The 60% accuracy reported here is calculated on a held* *‑* *out test set (n = 100) and represents the correct classification of participants into one of six RIASEC categories* *–* *a metric distinct from the domain* *‑* *level bivariate correlations (r) reported in previous literature.”*

**Discussion:** We will add a sentence noting that classification accuracy and bivariate correlation are not directly comparable, and that the PIMS model’s contribution is explanatory, not merely a statistical improvement – *“Because classification accuracy and bivariate correlation are not directly comparable, the PIMS model’s value lies not in surpassing the magnitude of domain* *‑* *level r values but in explaining the generative mechanisms that produce them.”*

**Response to Missing Prior Literature**

**POINT 3:** De Fruyt and Mervielde (1997)

 We note your observation that we did not reference a key facet-level study by De Fruyt and Mervielde (1997), which examined NEO-PI-R facet-level correlates of RIASEC interests.

**Our Response:** We acknowledge this omission. Our manuscript cites a later paper by the same authors (De Fruyt & Mervielde, 1999) but not the original 1997 facet study. We will add a citation and a discussion of how our results compare to theirs.

**How PIMS differs from De Fruyt & Mervielde (1997):** De Fruyt and Mervielde (1997) provided a valuable early examination of facet-level correlations, but they did not model the *dynamic sequences* of facets that we propose. They examined simple bivariate correlations between individual facets and interest scales. In contrast, the PIMS framework explicitly models the *interaction* of multiple facets in a predictive sequence (e.g., the combination of low Resilient, low Unguarded, and low Collaborative with high Thrill-seeking and high Competent to predict Investigative interests). The PIMS framework further proposes a qualitative shift: interests as emergent outcomes of personality dynamics, not as environmental matches.

**Our Revision:**

 We will add a paragraph to either the Introduction or Discussion: *“De Fruyt and Mervielde (1997) provided the first systematic examination of NEO-PI-R facet-level correlates of RIASEC interests, demonstrating that individual facets show differential associations with interest types. While foundational, their analysis focused on bivariate correlations. The PIMS framework extends this work by modelling the combined, interactive effects of multiple facets in motivational sequences, and by proposing that interests emerge from these dynamics rather than merely correlating with them.”*

**POINT 4: **Armstrong and Anthoney (2009)

 We also note that we missed the replication and extension study by Armstrong and Anthoney (2009), which used property vector fitting to map IPIP facets onto the RIASEC circumplex.

**Our Response:** We acknowledge this omission. We used Etzel, et al., 2025, a more recent study - who also proposes a highly complex circumplex argument to unpack the similar conclusions we would have derived from Armstrong and Anthoney (2009). However, we agree, including Armstrong and Anthoney (2009) would have further reinforced the point we made using Etzel et al., 2025.

**How PIMS differs from Armstrong & Anthoney (2009):** Armstrong and Anthoney (2009) map facet-level correlates onto a pre-existing structural model of interests (the RIASEC circumplex). While their analysis asks: where in the interest space do personality facets falls, our PIMS framework asks a different question: how do specific combinations of facets generate interests as emergent outcomes? Also, Armstrong and Anthoney (2009) provide a spatial mapping while our PIMS framework provides a *temporal and mechanistic* model: sequences of trait configurations predispose individuals toward specific vocational pathways. The PIMS Atlas (Figures 5-6) is designed to operationalise this through cosine similarity matching - a dynamic alignment of whole profiles, not a static mapping of individual facets onto a circumplex.

**Our Revision:**

 To further factor in your advice, we’ll add the following to our Discussion:

*“Armstrong and Anthoney (2009) demonstrated that Openness facets consistently map onto the Artistic region of the RIASEC circumplex, and Extraversion facets onto the Social-Enterprising region - findings that our facet-level regression results corroborate. However, the PIMS framework moves beyond spatial mapping. By modelling the interactive effects of facets (e.g., the suppression and compensation effects we identified in Investigative and Conventional profiles), and by proposing that interests emerge from these dynamic sequences, we provide a generative account of interest formation. The PIMS Atlas extends this by enabling dynamic profile matching, rather than static facet mapping.”*

**Response to Categorical vs. Continuous Issue**

**Point 5: “Predicting discrete Holland categories from continuous facets is ironic”**

 Here you note that it is “ironic” to predict discrete Holland categories from continuous personality facets, especially since RIASEC scores are continuous and practitioners use three-letter codes.

**Our Response:** We fully agree. This is an inherent limitation of using the RIASEC typology as our criterion, and we acknowledge it *explicitly* in the Limitations section (Section 4.2):

*“There is an inherent irony in our methodological choice. To demonstrate the predictive power of our personality-based model, we used the RIASEC interest categories as our target - the very typology our framework seeks to move beyond.”*

 We also note that the true promise of PIMS lies in eventually replacing broad typological outcomes with more nuanced, personality-defined pathways. The PIMS Atlas (Figures 5-6) is designed to generate continuous congruence scores (cosine similarity coefficients), not categorical assignments.

 Based on your guidance, we will emphasise this point further.

**Our Revision:**

**Limitations:** Here we’ll add a sentence as follows: *“The use of a single dominant RIASEC category as the dependent variable is a simplification. In practice, individuals present with profiles across multiple types (e.g., three-letter codes). The PIMS framework is designed to eventually operate with continuous profile matching via cosine similarity, as illustrated in Figure 5.”*

**Response to Causal and Emergence Claims**

**Point 6:** “The claim that facets cause interests is a metaphysical assumption”

 We note that you question the claim that personality facets represent “dynamic motives” that “cause” vocational interests, suggesting this is a “metaphysical assumption.” Also, that you recommend either emphasising the hypothetical nature of this claim or reviewing the Block vs. Mendelsohn debate on emergence.

**Our Response:**

 You raise a valid point about the need for caution in making causal claims from any non-experimental data. Here, we wish to clarify two matters: first, the evidentiary basis of our study; second, the theoretical lineage of our configural claims.

**Regarding Evidence:**

 Regarding the emergence debate: Thank you for directing our attention to the Block–Mendelsohn debate. We recognise that the PIMS framework is not neutral between them. Rather, PIMS explicitly aligns with Block’s emergentist view (Block & Ozer, 1982) - which serves as a clear prolepsis (intellectual anticipation) of our framework. Block and Ozer argued that configurations of personality traits produce emergent properties not reducible to the sum of individual traits, because traits interact and alter each other’s meaning. This is precisely what PIMS proposes: vocational interests are emergent outcomes of facet-level motivational sequences, not additive combinations of domain (or even facet) scores. Our identification of *suppression effects* (e.g., low Resilience intensifying intellectual drive in Investigative profiles) and *compensation effects* (e.g., high emotional stability offsetting low cautiousness in Conventional profiles) exemplifies the configural, non-additive logic Block championed.

 Conversely, Mendelsohn, Weiss, and Feimer (1982) argued that configural effects can be adequately explained by additive linear combinations of traits, favouring parsimony until strong evidence for non-additivity emerges. Theoretically, Mendelsohn et al.’s assumption of additive linear combinations is problematic because linearity and conjoint additivity are mathematical criteria that assume interval‑scale measurement and independence - conditions that Likert‑scale count data do not satisfy. Thus, their operationalisation of ‘additive linear combinations’ is not as straightforward or assumption‑free as they imply. While we respect their parsimony principle, the entire motivation for the PIMS framework is that additive domain-level models have plateaued ( *r* ≈ .48), and that the field requires a configural, emergentist approach to move beyond description toward explanation. For this reason, Block’s position is more aligned with the theoretical foundation of PIMS.

 We do acknowledge, however, that our cross-sectional predictive data cannot definitively adjudicate between Block and Mendelsohn. We agree that longitudinal mediation studies are required to test whether configural effects (e.g., the specific three‑facet sequence for Investigative interests) statistically mediate the development of vocational interests over and above additive combinations - a point we have explicitly emphasised in our Future Research Directions section.

**Our Revision:**

**Abstract:** Change “causative framework” to “explanatory framework”.

**Discussion (new paragraph we will add):**

*“The claim that interests emerge from facet-level trait configurations - rather than merely correlating with them - is a hypothesis, not a demonstrated fact. Our study demonstrates that a multinomial model using 30 facet-level traits can predict primary RIASEC categories with 60% accuracy on a held-out test set, substantially exceeding chance (16.7%). This predictive power is a necessary condition for, but not proof of, a generative causal relationship. The PIMS framework aligns with Block & Ozer’s (1982) argument that configurations of traits can produce emergent properties not reducible to individual traits (a prolepsis of our approach), as instantiated by the suppression and compensation effects we identified in Investigative and Conventional profiles. We acknowledge the alternative additive view (e.g., Mendelsohn, Weiss, & Feimer, 1982), but note that the field’s plateau at domain* *‑* *level correlations motivates a configural, emergentist direction. Our predictive model cannot definitively adjudicate between these perspectives. Longitudinal mediation analyses are required to test whether facet-level configurations mediate interest development beyond additive effects.”*

**Future Research Directions:** Here we already make the point explicitly in our paper namely, *“To progress from identifying correlations to explicitly testing the proposed generative mechanisms, future studies should employ formal mediation analysis. For instance, longitudinal studies could assess whether specific facet-level traits (e.g., low Resilience and low Sociability) statistically mediate the development of Investigative interests over time, thereby providing causal pathway evidence for the PIMS sequences proposed in this study.”*

**Response to Data Availability**

**Point 7: **“The raw data does not seem to be available”

 We note that you point out that “the raw data itself does not seem to be available,” despite the authors’ claim.

**Our Response:** The raw data are available. We have included a Data Availability Statement on page 20 of the manuscript, citing a Figshare repository.

**Our Revision:**

**Data Availability:** Townsend, G. C., & Webb, P. (2026). Underlying Data - Decoding Decisions: The Predictive Power of PIMS [Data set]. Figshare. https://doi.org/10.6084/m9.figshare.c.8271115
